# Seasonal prediction of the boreal winter stratosphere

**DOI:** 10.1007/s00382-021-05787-9

**Published:** 2021-05-18

**Authors:** Alice Portal, Paolo Ruggieri, Froila M. Palmeiro, Javier García-Serrano, Daniela I. V. Domeisen, Silvio Gualdi

**Affiliations:** 1 Department of Earth and Environmental Sciences, Università degli Studi di Milano-Bicocca, Milan, Italy; 2 Laboratoire de Météorologie Dynamique/IPSL, Ecole Normale Superieure, PSL Research University, Sorbonne Université, École Polytechnique, IP Paris, CNRS, Paris, France; 3 Department of Physics and Astronomy, University of Bologna, Bologna, Italy; 4 Fondazione Centro Euro-Mediterraneo sui Cambiamenti Climatici (CMCC), Bologna, Italy; 5 Group of Meteorology, Universitat de Barcelona (UB), Barcelona, Spain; 6 Institute for Atmospheric and Climate Science, ETH Zürich, Zurich, Switzerland; 7 Istituto Nazionale di Geofisica e Vulcanologia (INGV), Bologna, Italy

**Keywords:** Seasonal predictions, Stratosphere, Lower-stratosphere wave activity, Meridional eddy heat flux, Sudden stratospheric warmings

## Abstract

The predictability of the Northern Hemisphere stratosphere and its underlying dynamics are investigated in five state-of-the-art seasonal prediction systems from the Copernicus Climate Change Service (C3S) multi-model database. Special attention is devoted to the connection between the stratospheric polar vortex (SPV) and lower-stratosphere wave activity (LSWA). We find that in winter (December to February) dynamical forecasts initialised on the first of November are considerably more skilful than empirical forecasts based on October anomalies. Moreover, the coupling of the SPV with mid-latitude LSWA (i.e., meridional eddy heat flux) is generally well reproduced by the forecast systems, allowing for the identification of a robust link between the predictability of wave activity above the tropopause and the SPV skill. Our results highlight the importance of November-to-February LSWA, in particular in the Eurasian sector, for forecasts of the winter stratosphere. Finally, the role of potential sources of seasonal stratospheric predictability is considered: we find that the C3S multi-model overestimates the stratospheric response to El Niño–Southern Oscillation (ENSO) and underestimates the influence of the Quasi–Biennial Oscillation (QBO).

**Supplementary Information:**

The online version supplementary material available at 10.1007/s00382-021-05787-9.

## Introduction

The dynamical evolution of the stratosphere is driven by different processes. Radiative absorption by ozone causes differential warming in the vertical due to the increased absorption at higher levels, while a meridional gradient is generated by the latitudinal variations in incoming solar radiation, following the seasonal cycle (Andrews et al. 1987). Hence, the stratosphere is dominated by vertical stability and strong seasonality. In the winter hemisphere, the strong mid-latitude meridional temperature gradient supports an intense westerly wind, commonly known as the *stratospheric polar vortex* (SPV). Its daily to interannual variability is modulated by the planetary waves resulting from the interaction between upward tropospheric wave activity and the lower stratosphere (Sjoberg and Birner 2014); these propagate into the stratosphere, grow, break and eventually decelerate the zonal-mean flow (Andrews et al. 1987). The boreal SPV is slower and more variable than its austral counterpart, as a result of the stronger wave activity induced by large orographic structures (Leovy and Webster 1976) and land-sea thermal contrast (Chen and Trenberth 1988; Garfinkel et al. 2020). In the Northern Hemisphere, strong anomalies in the stratospheric circulation are known to propagate towards lower levels and to influence mid-latitude surface weather and climate (see Kidston et al. 2015, for a review).

After the seminal works by Baldwin and Dunkerton (1999, 2001), much attention has been brought to the link between the stratosphere and troposphere, in terms of dynamics and predictability. Various time scales have been explored, from subseasonal to centennial (Kidston et al. 2015), with emphasis given to *sudden stratospheric warming* events—SSWs (Matsuno 1971; Baldwin et al. 2021). These are abrupt disruptions of the SPV that can affect the tropospheric circulation for up to two months (e.g. Sigmond et al. 2013). Since deterministic predictability of SSWs does not extend beyond 10–20 days (Tripathi et al. 2015; Domeisen et al. 2020a), no deterministic skill is expected above such range. Nonetheless, probabilistic predicability of SSWs is found for time scales of weeks (Domeisen et al. 2020b; Garfinkel and Schwartz 2017) to seasons (Scaife et al. 2016; Taguchi 2018). To our knowledge, this study provides the first multi-model evaluation of probabilistic prediction skill for SSWs for the seasonal range; our new approach is based on category SSW forecasts.

The simulation of the seasonal evolution of the stratosphere depends on the representation of a range of slowly varying processes. From reanalysis data and model studies, we learn that the stratosphere is affected by tropical tropospheric forcing associated with ENSO (see Domeisen et al. 2019, for a review) and by surface forcing in mid-latitude and polar regions, e.g. induced by Arctic sea-ice extent (see Cohen et al. 2014, for a review) and Eurasian snow cover (see Henderson et al. 2018, for a review). The QBO (Baldwin et al. 2001) in the tropical stratosphere is also known to influence the extratropical stratosphere through the so-called Holton–Tan effect ( Holton and Tan 1980; Anstey and Shepherd 2014, for a review). In forecast systems the representation of these sources of variability and of their interaction with the stratosphere may modulate the strength of the winter SPV and, consequently, the SPV-forced tropospheric circulation. Additionally, there is evidence that a high model top and high vertical resolution favour a realistic simulation of troposphere–stratosphere coupling (e.g. Gerber and Polvani 2009; Charlton-Perez et al. 2013), hence the interaction between tropospheric seasonal sources of variability and the stratosphere (Butler et al. 2016). Seasonal predictions of near-surface climate in the mid latitudes are indeed expected to improve with a well-resolved stratosphere (e.g. Folland et al. 2012; Smith et al. 2012), as appears to be the case for the North Atlantic Oscillation (Scaife et al. 2014a, 2016) and generally over the North Atlantic (Domeisen et al. 2015; Butler et al. 2016), northern Eurasia and North America (Jia et al. 2017). Likewise, some works indicate that seasonal prediction benefits from initialising the stratosphere (Stockdale et al. 2015; O’Reilly et al. 2019; Nie et al. 2019).

While troposphere–stratosphere interaction has been analysed extensively, few studies have explored its dynamics in the seasonal prediction context. Note that process-oriented assessment (e.g. Lee et al. 2020a) is not a common practice in forecast verification, which is usually focused on skill scores of direct outputs. Recent efforts have been made to investigate the dynamics of tropospheric teleconnections (e.g. Molteni et al. 2015; Scaife et al. 2017) or to analyse the role of the stratosphere in subseasonal forecasting (e.g. Domeisen et al. 2020a, b, and references therein); the latter under the framework of SNAP—Stratospheric Network for the Assessment of Predictability, an activity of SPARC/WCRP (https://www.sparc-climate.org/activities/assessing-predictability). The present study fills that gap by assessing stratospheric wave–mean-flow interaction in seasonal forecast systems. Additionally, we update the analysis of prediction skill in the boreal winter stratosphere (cf. Maycock et al. 2011; Butler et al. 2016) for the high-top C3S seasonal forecast systems.

To summarise, the purpose of this work is to describe how seasonal forecast systems simulate and predict the stratosphere, and how they represent the link between the SPV and LSWA, where the latter is generally attributable to upward-propagating planetary Rossby waves. In particular, we address the following questions: Do seasonal forecast systems reproduce a realistic variability of the Northern Hemisphere winter stratosphere, and can they predict the winter SPV and the number of SSWs per winter? (Sect. 3.1) Secondly, how is the variability and prediction skill of the SPV connected to LSWA? (Sect. 3.2 and 3.3) And where does the seasonal skill arise from? (Sect. 3.3). A comprehensive interpretation of the results is given in Sect. 4, while the main findings and perspectives are outlined in Sect. 5.

## Methods

### Data

In this work retrospective forecasts (hindcasts) from five state-of-the-art climate models are analysed and compared with ERA-Interim reanalysis. These are five European seasonal prediction systems taking part in the C3S: Euro-Mediterranean Center on Climate Change (CMCC system 3: Sanna et al. 2017), Météo-France (MF system 6: Dorel et al. 2017; Batté and Déqué 2016), European Centre for Medium-Range Weather Forecasts (ECMWF SEAS5: Johnson et al. 2019), Deutscher Wetterdienst (DWD system 2: Baehr et al. 2015; Jungclaus et al. 2013; Stevens et al. 2013), and the UK Met Office (UKMO GloSea5: MacLachlan et al. 2015). The hindcasts are initialised on November 1st, except for MF and UKMO with start dates around the beginning of November, over the period 1993–2016. Further details, including model resolution and ensemble size, are listed in Table 1. Daily data of zonal wind (10-to-100 hPa), meridional wind and temperature (100 hPa) are obtained for all available ensemble members from November to April.

**Table 1:** General description of the seasonal prediction systems contributing to the C3S multi-model.

Models	Resolution	Initial Conditions	Ensemble Size
**CMCC** (system 3)	$1^\circ$ lat/long 46 L	1st November	40 members
**MF** (system 6)	TL359 91 L	20th, 25th October 1st November	2$\times$12 members 1 member
**ECMWF** (SEAS5)	T_CO_319 91 L	1st November	25 members
**DWD** (system 2)	T127 95 L	1st November	30 members
**UKMO** (GloSea5, system 13)	N216 95 L	25th October 1st, 9th November	7 members per start date

For vertical resolution we indicate the number of vertical levels (L)

Indices for ENSO, the QBO, and Arctic sea-ice extent are taken from https://www.ncdc.noaa.gov, Eurasian snow cover from https://climate.rutgers.edu. Specifically, ENSO is measured by the Niño 3.4 index in December–January–February (DJF), the QBO by equatorial zonal-mean zonal winds at 30 hPa in DJF. Arctic sea-ice extent and Eurasian snow cover are considered in October-November (ON).

### Indices of stratospheric variability

**Fig. 1:**
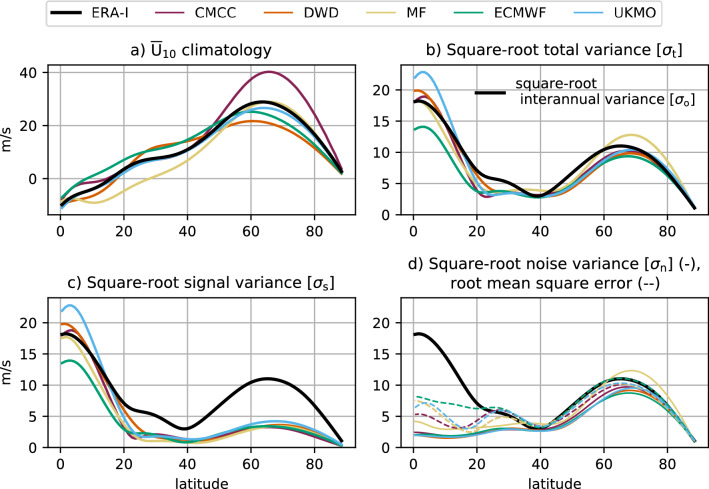
Climatology and variability of ${\overline{U}}_{10}$ in the Northern Hemisphere winter (DJF). **a** Climatology. **b** Square root of total variance and **c** square root of signal variance. **d** Square root of noise variance (solid lines) and root mean square error of the ensemble-mean (dashed lines). For reference, the black line in **b**–**d** stands for the interannual variance from ERA-Interim

**Fig. 2:**
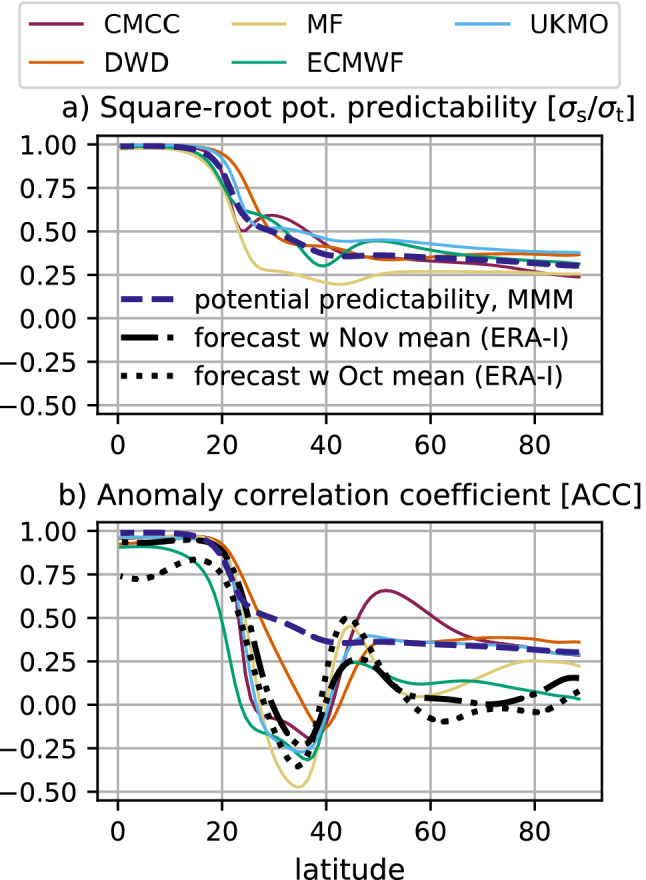
Predictability and prediction skill of ${\overline{U}}_{10}$ in the Northern Hemisphere winter (DJF). **a** Square root of potential predictability (PP). **b** Anomaly correlation coefficient (ACC). The ACC for empirical forecasts obtained by persisting observed monthly anomalies of October and November are shown with black dotted and dash-dotted lines, respectively. The mean multi-model (MMM) PP is represented by the dark-blue dashed line

**Fig. 3:**
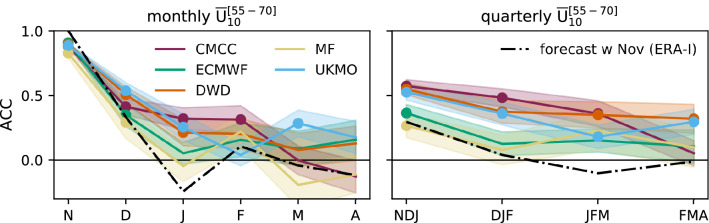
Anomaly correlation coefficient (ACC) of ensemble-mean monthly and quarterly ${\overline{U}}_{10}^{[55-70]}$ anomalies; shading indicates the standard deviation. Results for empirical forecasts based on the persistence of the observed November anomaly are represented by the black dot-dashed line. Significant positive ACC at the 95% confidence level, using bootstrap resampling with 1000 realisations (Appendix B), is shown by full coloured circles

**Fig. 4:**
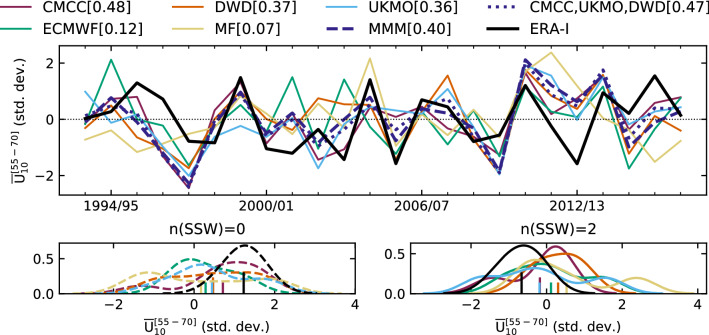
(Top) Time series of anomalous ${\overline{U}}_{10}^{[55-70]}$ in DJF from ERA-Interim (black line) and the ensemble-mean forecasts (coloured lines). Both are standardised in order to allow for a direct comparison. The multi-model ensemble-mean (MMM) and the average over CMCC, UKMO and DWD are shown in dark blue by dashed and dotted lines, respectively. The corresponding ACC is shown in parenthesis. (Bottom) Distribution of standardised DJF ${\overline{U}}_{10}^{[55-70]}$ anomalies for winters with no observed SSW (left, dashed lines) and with 2 observed SSWs (right, full lines). A Gaussian Kernel Density Estimate with bandwidth equal to $1/2 \, \sigma$ is used to compute the distributions. The mean value of each distribution is indicated by a short vertical line at the x-axis

**Fig. 5:**
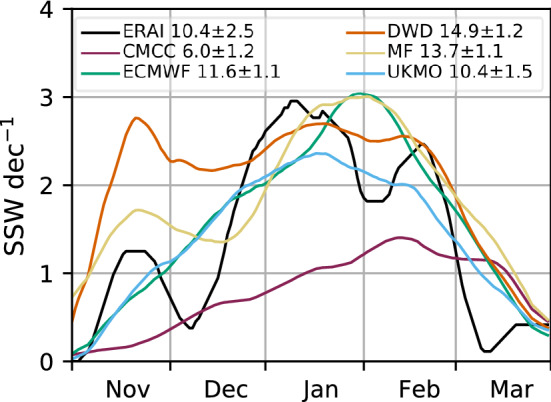
November to March seasonal distribution of SSWs per decade in a [− 10,+ 10]-day window around the SSW date for ERA-Interim and the forecast systems, with SSWs selected using the 55_70N definition (see Sect. 2.2). The average SSW frequency per decade is indicated next to each label. Time-series are smoothed with an 11-day running mean

**Fig. 6:**
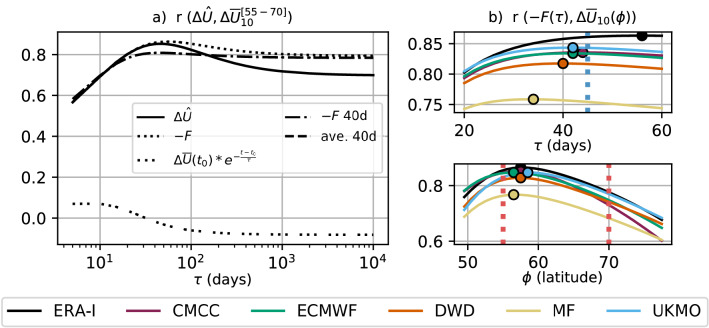
**a** Correlation between different estimates of the wind anomaly and the actual anomaly at 10 hPa, 55–70$^\circ$ N (see vertical red lines in **b**) for different values of the radiative relaxation time scale ($\tau$) in the anomaly estimate, using daily DJF data (ERA-Interim). The solid line is the correlation with $\varDelta {\hat{U}}$ (Eq. (2)) including stratospheric initialisation at time $t_0$ (triple dots), the dotted line is obtained by considering only $-F$ (heat-flux integral in Eq. (2)) with lower boundary fixed to the 1st of November (9th of November for 7 members in UKMO), while the dash-dotted line is produced by calculating *F* over a 40-day moving window. The horizontal dashed line is a commonly used 40-day average (e.g. Polvani and Waugh 2004, note that this corresponds to the limit $\tau \rightarrow \infty$ for *F* 40d). The x axis is displayed with logarithmic scaling. **b** Correlation between daily DJF values of $-F$ and $\varDelta {\overline{U}}_{10}$ as a function of the radiative relaxation time scale ($\tau$, top) and of latitude ($\phi$, bottom) for ERA-Interim and the forecast systems, with circles indicating the maximum. In the top panel $F(\tau )$ is computed with $\tau$ from 20 to 60 days; $\varDelta {\overline{U}}_{10}$ as in the left panel. In the bottom panel $\varDelta {\overline{U}}_{10}(\phi )$ is the average $\varDelta {\overline{U}}_{10}$ in the 5-degree latitude band around $\phi$ and $F(\tau )$ is computed with $\tau _{10}=45$ days (see vertical blue line)

**Fig. 7:**
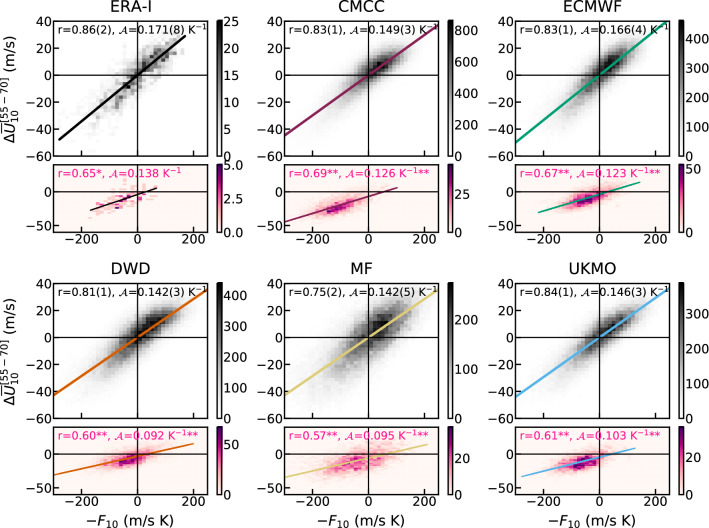
Two-dimensional density histograms between $-F_{10}$ (see Eq. (2)) and $\varDelta {\overline{U}}_{10}^{[55-70]}$ for ERA-Interim and the forecast systems. Grey histograms show the analysis using daily data over DJF; coloured histograms are constructed with DJF days preceding SSWs, i.e. in the [− 6,0]-day window centered in the event (SSWs according to the 55_70N criterion, see Sect. 2.2). We estimate the correlation (r) and slope (${\mathcal {A}}$) between the two variables in each plot. The uncertainty $\sigma _r$ or $\sigma _{{\mathcal {A}}}$ on the last figure of a coefficient is indicated in parenthesis (see Sect. 2.2 for details on the calculation). SSW coefficients significantly different from the non-SSW at 99% (95%) confidence are indicated by ** (*), as from a bootstrap on r and ${\mathcal {A}}$, computed with the SSW sample size but over non-SSW days, i.e. outside the [− 10,10]-day window centered in SSWs

**Fig. 8:**
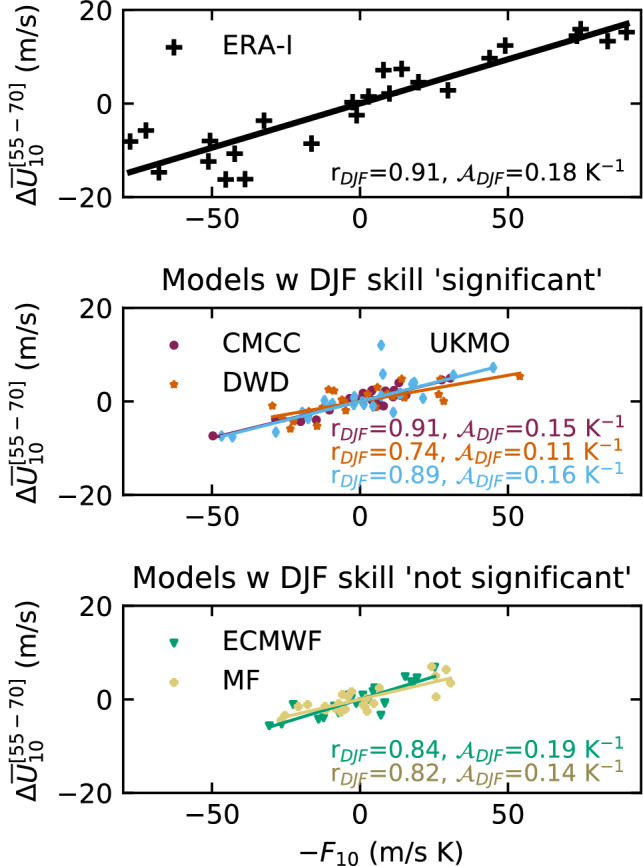
Scatter plot between DJF averages of $-F_{10}$ (see Eq. (2)) and $\varDelta {\overline{U}}_{10}^{[55-70]}$ for ERA-Interim (top) and the ensemble-mean forecasts (middle, bottom). Models are arranged depending on their prediction skill for the SPV wind (Figs. 2b, 3). We estimate the correlation ($\hbox {r}_{DJF}$) and slope (${\mathcal {A}}_{DJF}$) between the two variables

Consistently with the prevailing zonal symmetry of the stratospheric circulation, the state of the Northern Hemisphere stratosphere is diagnosed by zonal-mean zonal winds at 10 hPa, hereafter ${\overline{U}}_{10}$. We choose the 10-hPa level to characterise the variability of the mid stratosphere—the same level is often chosen, for instance, to identify SSW events (Charlton and Polvani 2007; Butler et al. 2015). The strength of the SPV is here defined as the average of ${\overline{U}}_{10}$ between 55 and 70$^\circ$ N, from now on ${\overline{U}}_{10}^{[55-70]}$; alternative definitions of SPV intensity, for instance ${\overline{U}}_{10}$ at 60$^\circ$ N, do not qualitatively affect the results presented in Figs. 1, 2, 3, 4, 5, 6, 7 and 8 (not shown). The ${\overline{U}}_{10}^{[55-70]}$ definition has been preferred as, in principle, it mitigates the role of model biases in the position of the strongest 10-hPa winds, evident from Fig. 1a.

SSWs are identified through two different definitions: the ${\overline{U}}_{10}$ inversion in at least one of the latitudes between 55 and 70$^\circ$ N (Palmeiro et al. 2015) and the traditional ${\overline{U}}_{10}$ inversion at 60$^\circ$ N (Charlton and Polvani 2007), henceforth “55_70N” and “60N” definition, respectively. In both cases, distinct SSW events must be separated by at least 21 days of westerly winds and *final warmings*, which mark the transition to easterly summer winds—here defined as ${\overline{U}}_{10}$ inversions at all considered latitudes for at least 21 days, are discarded. As explained in Palmeiro et al. (2015), the 55_70N definition is convenient because it is not sensitive to biases in the latitude of the SPV, and, despite the higher number of SSWs compared with the 60 N definition (e.g. Fig. S4), the downward propagation signal is similar in intensity and extent.

On seasonal and interannual time scales the winter variability of the SPV is induced by vertical propagation of planetary Rossby waves from the troposphere (Andrews et al. 1987; Newman et al. 2001); wave generation internal to the stratosphere also occurs (Scott and Polvani 2004; Birner and Albers 2017; Boljka and Birner 2020). It is mainly low-frequency, low-wavenumber planetary waves that propagate through the stratosphere, break in the strong mid-stratospheric zonal flow and slow down the vortex by transferring easterly momentum to the westerly winds (Andrews et al. 1987; Haklander et al. 2007). The mid-latitude, 100-hPa meridional eddy heat flux (henceforth “eddy heat flux” or $[v^*T^*]$, where $^*$ is the deviation from the zonal mean and [..] denotes the area-weighted average between 40 and 80$^\circ$ N) is used to diagnose LSWA (Scott and Polvani 2004).

Hinssen and Ambaum (2010) developed an analytical relation between stratospheric potential vorticity and 100-hPa meridional eddy heat flux (Eq. (9) in their article). Here we adapt it to link ${\overline{U}}_{10}$ and the eddy heat flux, by inverting a quasi-geostrophic scaling between wind and potential vorticity—namely $\varDelta {\overline{q}} \approx - \frac{\partial \varDelta {\overline{U}}}{\partial y}$, with *q* quasi–geostrophic potential vorticity and $\varDelta$ indicating the anomaly with respect to climatology. Therefore, we derive the following approximation for $\varDelta {\overline{U}}$ at a generic latitude $\phi$ and pressure-level *p* in the extratropics

1 $$\begin{aligned} \varDelta {\overline{U}}(\phi ,p,t)&\approx \varDelta {\hat{U}}(\phi ,\tau _p, t), \end{aligned}$$

2 $$\begin{aligned} \varDelta {\hat{U}}(\phi ,\tau _p,t)\equiv& \varDelta {\overline{U}}(\phi ,p,t_0) \; e^{-(t-t_0)/\tau _p} - {\mathcal {A}} \, F(\phi ,\tau _p,t)\nonumber \\\equiv& \varDelta {\overline{U}}(\phi ,p,t_0) \; e^{-(t-t_0)/\tau _p} \nonumber \\&- {\mathcal {A}} \, \int _{t_0}^t \varDelta [v^*T^*](t^\prime ) \; e^{-(t-t^\prime )/\tau _p} \mathrm {d}t^\prime , \end{aligned}$$

where $\tau _p$ is a radiative time scale which depends on the pressure level considered, ${\mathcal {A}}$ is a constant expressed in units of $K^{-1}$. The initial condition $\varDelta {\overline{U}}(t_0)$ decays exponentially at a rate of $1/\tau _p$. Equivalently, the importance of the eddy-heat-flux anomaly decays as the interval between *t*, the time when the wind anomaly is evaluated, and $t^\prime$, an earlier time step, increases. *F*(*t*) is defined as the time integral on the eddy-heat-flux anomalies; in our case the lower boundary $t_0$ is set to the initialisation time of the seasonal forecast and the corresponding day of the year in the reanalysis. Details for the derivation of Eq. (2) are given in Appendix A, while in Sect. 3.2 we show that for our problem it is convenient to consider $\varDelta {\hat{U}}\sim -{\mathcal {A}} F$.

In the analysis on the coupling between LSWA and the SPV, we compare the anomaly of the vortex wind at 10 hPa with the integral of 100-hPa eddy heat flux. A reasonable radiative time scale for the 10-hPa level, i.e. $\tau _{10}=45$ days, is used to estimate *F* in the mid stratosphere ($F_{10}\equiv F(\tau _{10})$). Correlation (**r**, Pearson’s definition) and slope ($\sim {\mathcal {A}}$ in Eq. (2)) obtained from the linear regression of the two variables are used to measure the importance of wave activity for the variability of the extratropical stratosphere and the magnitude of wave forcing on the mid-stratospheric mean flow, respectively. In particular, **r**$^2$ indicates the proportion of vortex-wind variance that is explained by LSWA. An estimate of uncertainty for **r** and ${\mathcal {A}}$ is calculated using bootstrap resampling. Full time series of daily ($F_{10}$, $\varDelta {\overline{U}}_{10}^{[55-70]}$) points, labelled by year and ensemble member, are randomly extracted to give a bootstrap sample of $n_m \times n_y$ points, where $n_m$ is the model ensemble size and $n_y$ the number of years. In this way the internal time correlation is included (block-bootstrap method, Carlstein (1986)). The procedure is repeated 1000 times, each giving a possible outcome of **r** and ${\mathcal {A}}$; from the resulting distributions we compute the standard deviations $\sigma _r$ and $\sigma _{\mathcal {A}}$, used to measure uncertainty.

### Forecast verification metrics and statistical methods

A large portion of the analysis assesses the performance of stratospheric forecasts taken from a multi-model set of ensemble seasonal hindcasts. Let $m_{y,j}$ be a variable of the forecast (e.g. the DJF average of a model variable) for year *y* and ensemble member *j*, and let ${\mathbb {E}}[\cdot ]_{x}$ denote the mean operator over the index *x*, then the ensemble mean and spread in year *y* are $M_y= {\mathbb {E}} \left[ m_{y,j}\right] _{j}$ and $S_y= \sqrt{{\mathbb {E}}\left[ (m_{y,j}-M_y)^2\right] _j}$, the model climatology is $C = \left[ M_{y}\right] _{y}$. *Total variance*, *signal variance*, i.e. interannual variance of the ensemble mean, and *noise variance*, i.e. mean variance around the ensemble mean or square of the mean *spread*, are respectively

3 $$\begin{aligned} \sigma ^2_t&= {\mathbb {E}} \left[ (m_{y,j}-C)^2 \right] _{y,j}, \end{aligned}$$

4 $$\begin{aligned} \sigma ^2_s&= {\mathbb {E}}\left[ (M_y-C)^2 \right] _y,\end{aligned}$$

5 $$\begin{aligned} \sigma ^2_n&= {\mathbb {E}} \left[ S_y^2\right] _y. \end{aligned}$$

Note that *total variance*, $\sigma ^2_t$ , is equal to the sum of $\sigma ^2_s$ and $\sigma ^2_n$.

The *observed interannual variance* is computed with Eq. (4) replacing $M_y$ with the corresponding reanalysis value $O_y$ and C with the observed climatology $C_O$ over the forecast period. The *root-mean-square error* is defined by

6 $$\begin{aligned} \text {RMSE}= \sqrt{ {\mathbb {E}}\left[ \left( (M_y-C) - (O_y - C_O)\right) ^2 \right] _y }. \end{aligned}$$

Since a single ensemble member of an ideal forecast system is a possible outcome of the atmospheric system, then $\sigma _n$ should tend to the RMSE in the limit of large ensemble size (Fortin et al. 2014) or in well-calibrated forecast systems (Doblas-Reyes et al. 2013). However, forecast systems may be underdispersive/overconfident ($\sigma _n<\text {RMSE}$) or overdispersive/underconfident ($\sigma _n>\text {RMSE}$).

Seasonal predictability is analysed using *potential predictability*, whereas seasonal prediction skill using *anomaly correlation coefficient*

7 $$\begin{aligned} \text {PP} = \frac{\sigma ^2_s}{\sigma ^2_t}, \qquad \text {ACC} = \frac{\rho _{so}}{\sigma _s \cdot \sigma _o} \end{aligned}$$

where $\rho _{so}$ indicates the covariance between ensemble-mean and observational anomalies. The PP, predictable fraction of model variance, is generated by slowly varying drivers of atmospheric variability (Smith et al. 2012), and gives an upper bound for the skill of the forecast system. If PP=1 then variability is completely determined by predictable factors (Eade et al. 2014), while PP=0 characterises a climate that is dominated by unpredictable noise. For an extended description of the forecast quality assessment we refer to Von Storch and Zwiers (1999) and Jolliffe and Stephenson (2012).

The statistical significance of positive ACC and of linear regression coefficients is evaluated with two alternative methods. In the first case, we calculate a threshold value for ACC (regression coefficients) using one-(two-)tailed Student’s t test; in the second case (ACC in Fig. 3 and regression in Fig. 11), we determine the significance of each value through a *bootstrap* procedure that gives an estimate of the unknown distribution (see Appendix B for details).

Probabilistic skill of SSWs occurring in winter (DJF) is evaluated using the Brier Skill Score (BSS, Wilks 2006; Jolliffe and Stephenson 2012). Specifically, BSS assesses the ability of an ensemble forecast to predict years with a number of events below normal (Bn), above normal (An) or in the normal range (n). In ERA-Interim and four forecast systems (MF, ECMWF, DWD, UKMO), over 1993/94-2016/17, normal conditions are given by $\sim$1 SSW per winter; CMCC simulates a low number of SSWs (Fig. 5), with the normal range close to 0 SSW per winter. Hence, with the exception of CMCC, binary outcomes are considered on the basis of three categories: normal conditions are 1 SSW per winter, Bn (An) indicates 0 ($\ge$ 2) SSWs per winter. Note that the terciles depend on the hindcast/verification period and on the model considered. The mean square error of the probability forecast (Brier Score, BS) is used to compute the BSS for each of the three categories:

8 $$\begin{aligned} BS ={\mathbb {E}}\left[ (f - o)^2 \right] _{y}, \qquad \text {BSS}=\ 1 - \frac{\text {BS}_{adj}}{\text {BS}_{cl}} \end{aligned}$$

where the observed probability *o* is assigned 1 if the category was actually observed and 0 if it was not observed; the forecast probability *f* is the fraction of ensemble members predicting the chosen category. In BSS, the forecast BS is compared with a reference Brier Score ($\hbox {BS}_{cl}$) that is calculated by substituting *f* with $p_{cl}$, the observed climatological probabilities (0.29/0.54/0.17 for Bn/n/An). Alternatively, for comparisons with different verification periods, uniform probabilities are considered, i.e. 0.33/0.33/0.33.

Besides, the original BS is known to be unfair because it favours ensembles that are sampled from overconfident distributions and also fails to account for finite ensemble size (Fricker et al. 2013; Ferro 2014). These issues are overcome by following Ferro’s definition of an adjusted, unbiased Brier Score as: $\hbox {BS}_{adj}$ = BS - ${\mathbb {E}}\left[ f \cdot (1-f) \right] _{y} / (n_m -1)$, where $n_m$ is the ensemble size. Note that a perfect forecast has BSS=1 (BS=0), while a forecast poorer than that based on climatology gives negative BSS. The statistical significance of BSS is assessed with a 95% confidence sign test (DelSole and Tippett 2014, 2016).

## Results

### Variability and predictability of the boreal winter stratosphere

We first consider the observed characteristics and model performance in boreal winter (DJF), i.e. 1 month after the November initialisation (Sect. 2.1). 10-hPa zonal-mean zonal wind (${\overline{U}}_{10}$) is analysed north of the Equator, using diagnostics such as climatology, total/signal/noise variance, root mean square error (Fig. 1), potential predictability and anomaly correlation coefficient (Fig. 2). Three latitudinal regions can be identified on the basis of interannual variability and forecast quality: the *tropics* (0–20$^\circ$ N), with high variability and high skill, the *subtropics* (20–40$^\circ$ N), with low variability but no skill, and the *extratropics* (40–80$^\circ$ N), with moderate variability and some skill.

At low latitudes, the interannual variability of the stratosphere is largely controlled by the QBO (Baldwin et al. 2001), an oscillation of the tropical zonal-mean zonal wind with a period of about 28 months. The total variance is dominated by the signal variance (Fig. 1b, c), i.e. the ensemble-mean interannual variability, with very low levels of noise variance or ensemble *spread* (Fig. 1d), which translate into high potential predictability (Fig. 2a). Yet, the spread, smaller than RMSE (dashed lines in Fig. 1d), indicates that the forecast systems tend to be underdispersive or overconfident—this applies to the whole Northern Hemisphere. Note that overconfidence in forecast systems is a common failure in climate prediction (e.g. Doblas-Reyes et al. 2013). We highlight that UKMO overestimates the total/signal variance, while ECMWF underestimates it; the other forecast systems show variability consistent with ERA-Interim (Fig. 1b, c). Since the QBO is characterised by a long time scale and its phase is initialised correctly in models, the skill in capturing the evolution of the tropical winds is high, regardless of the level of accuracy of the QBO’s simulation throughout the whole winter season (Fig. 2; Scaife et al. (2014b); Stockdale et al. (2020)). Besides, the forecast systems outperform an empirical prediction based on the persistence of October anomalies (available at initialisation) in the deep tropics (Fig. 2b). This suggests that the models simulate and predict the slow dynamical-radiative relaxation rates in the lower stratosphere (Haynes 1998) better than simple damping processes. Nonetheless, the good performance in the tropical region does not necessarily lead to skill at predicting the QBO extratropical teleconnection (Butler et al. 2016), particularly beyond the first month after initialisation (Stockdale et al. 2020). Inter-model differences in variability and prediction skill of tropical winds above 100 hPa (Figs. S1, S2) are probably linked to the different QBO representation in the forecast systems (Garfinkel et al. 2018).

At subtropical latitudes, total and signal variance exhibit minimum amplitude, and all forecast systems remain overconfident (Fig. 1). In this case, ACC shows negative scores (Fig. 2b), far from the potential predictability (Fig. 2a), behaving similarly to empirical forecasts based on the persistence of October (lead 1) or November (lead 0) anomalies. Lack of predictive skill is apparent in the subtropics above 30 hPa (Fig. S2) and could be related to a poor representation of the QBO response in the region, as already found in CMIP5/6 climate models (Rao et al. 2020). This may affect the extratropical stratosphere by modifying the propagation of waves to the polar vortex.

At extratropical latitudes, the winter stratosphere is dominated by the variability of the SPV. The strongest winds in ERA-Interim are found at 63.5$^\circ$ N (Fig. 1a), whereas the models are slightly biased toward lower latitudes (ECMWF, DWD − 59.5$^\circ$ N, 60.5$^\circ$ N) or higher latitudes (MF, CMCC − 65.5$^\circ$ N). Also the strength of the SPV varies between forecast systems, with CMCC clearly overestimating and DWD underestimating the climatological maximum in reanalysis. The other three forecast systems (ECMWF, UKMO, MF) show weaker biases—weaker also than those in previous low-top generations ( Maycock et al. 2011, see also Fig. S1). Unlike the tropics, here the total variance (Fig. 1b) is dominated by the unpredictable noise variance (Fig. 1d), although with a contribution from the signal variance (Fig. 1c) which actually provides some potential predictability (Fig. 2a). In three out of the five forecast systems (CMCC, DWD, UKMO) prediction skill benefits from that available potential predictability (Fig. 2b). The other two forecast systems (ECMWF, MF) perform closer to the empirical predictions, indicating limited improvements with respect to forecasts based on the initial state (observed October anomaly, lead 1) or on the persistence of the observed, lead-0 November anomaly. The above suggests that some forecast systems are able to capture the dynamical mechanisms underlying SPV predictability beyond simple damping processes. Moreover, Fig. 3 indicates that the results in the seasonal range are not affected by the November performance (e.g. see ECMWF). We mention that CMCC is as skilful as models that have a much-better resolved stratosphere (e.g. DWD and UKMO, Table 1), even considering the positive bias in the SPV strength (Fig. 1a) and the low SSW frequency (Fig. 5).

Time series of anomalous winter ${\overline{U}}_{10}^{[55-70]}$ from ERA-Interim and the ensemble-mean forecasts are shown in the top panel of Fig. 4; standardisation is applied to aid comparisons. Individual forecast time series indicating the yearly ensemble spread are shown in Fig. S3, together with the observed winter occurrence of SSWs (55_70N definition, Sect. 2.2). In the bottom panels of Fig. 4 we relate the seasonal anomaly of SPV winds to the number of observed SSWs per winter, focused on years with no SSWs (left) and multiple SSWs (2 events; right). As expected, reanalysis anomalies are predominantly positive (negative) in years with 0 (2) SSWs, associated with a reinforced (weakened) polar vortex. The forecast anomalies, in spite of a large spread, show consistent results for no-SSW winters, with all forecast systems yielding a positive mean anomaly. On the contrary, no clear signal is found for winters with multiple SSW events.

SSWs are indeed known to impact the variability and predictability of the extratropical stratosphere (e.g. Scaife et al. 2016). To further diagnose the models’ performance in this regard, we first assess the simulation of SSW occurrence, and then the forecast quality. Four out of the five forecast systems (DWD, ECMWF, MF, UKMO) simulate a realistic SSW decadal frequency as compared with ERA-Interim (10.4 ± 2.5 SSW $\hbox {dec}^{-1}$), and, more importantly, broadly capture the observed intraseasonal cycle with a relative maximum of occurrence in mid-winter (January–February; Fig. 5). These results imply an improvement with respect to previous low-top generations (Maycock et al. 2011) and to up-to-date modelling systems taking part in CMIP5 (Horan and Reichler 2017). The exception is CMCC, exhibiting a strong vortex (Fig. 1a) and a reduced SSW frequency throughout the winter season (Fig. 5), with the SSW peak shifted towards late-winter (February–March) as commonly found in low-top models (Palmeiro et al. 2020). Conversely, DWD overestimates the occurrence of early SSWs, but at the same time presents surprisingly good SSW forecasts of the three November events during the hindcast period, predicted by more than 90% of the ensemble members (Fig. S4). This finding, however, relates to the subseasonal range and is out of the scope of our paper.

As mentioned in the Introduction, no deterministic (ensemble-mean) skill is expected for SSWs in seasonal climate prediction. However, this does not exclude the possibility of predicting some dynamical processes associated with the occurrence or absence of SSWs. This study tackles, for the first time, a proper forecast quality assessment to explore probabilistic (category) skill of SSWs in the C3S multi-model. The Brier Skill Score (BSS, Sect. 2.3) is used to evaluate model performance following a tercile-oriented approach, namely the skill in predicting a number of events below (Bn), equal to (n), or above (An) the normal frequency of SSWs per winter (DJF). In ERA-Interim and in the forecast systems the normal conditions are 1 SSW per winter (0 SSWs per winter in CMCC). Hence, the three categories considered here correspond to: 0 (Bn), 1 (n), and multiple ($\ge$ 2, An) SSWs occurring in winter; for CMCC, the binary outcomes are 0/$\ge$ 1 (n/An). To obtain BSS, the ensemble forecasts are compared with a reference prediction based on climatology. This is computed over the verification period 1993/94-2016/17, providing observed climatological probabilities of 0.29/0.54/0.17 (Bn/n/An). We also assess BSS using equal probabilities 0.33/0.33/0.33, for comparisons with scores computed over different verification periods. The results are displayed in Table 2A and show that the forecast systems are particularly skilful at predicting no-SSW winters (Bn), thereby years when the polar vortex is weakly perturbed and anomalously strong. This is consistent with the SPV-wind outcomes in winters with no observed SSW (Fig. 4, bottom-left panel). We stress that UKMO is the only model yielding significant probabilistic skill for all three categories.

**Table 2:** **﻿A**) Probabilistic skill in predicting the number of SSWs per winter (DJF), with events selected using the 55_70N definition (Sect. 2.2). Skill is evaluated with BSS for three categories: occurrence of SSWs below, equal to, and above normal conditions (Bn/n/An). BSS is obtained by comparing the dynamical forecasts to a prediction based on observed climatological probabilities or assuming equiprobability (values in parenthesis), while its confidence level is determined with a binomial test which considers successful years (BS < $\hbox {BS}_{ref}$) equiprobable to unsuccessful years (BS > $\hbox {BS}_{ref}$); see Sect. 2.3 for details. Note that for CMCC normal conditions correspond to zero SSWs per winter and $\hbox {BSS}_{Bn}$ cannot be calculated (n.c.) **B**) Deterministic skill, i.e. anomaly correlation coefficient (ACC), between ensemble-mean and reanalysis SPV-wind in DJF. Reanalysis SPV-wind is ${\overline{U}}_{10}^{[55-70]}$, model wind is estimated in three ways: (1) as for the reanalysis, (2) through $F_{10}$, integral over forecast anomalies of November-to-February eddy heat flux, and (3) same as (2) but considering forecast anomalies only in November; these produce (1) $\hbox {ACC}_{U}$, (2) $\hbox {ACC}_{F}$ and (3) $\hbox {ACC}_{F(N)}$, respectively. For each forecast system, we highlight in bold the best ACC score, excluding MF that yields low, non-significant values **C**) Correlation between $-F_{10,reg}$ and $\varDelta {\overline{U}}_{10}^{[55-70]}$ (DJF ensemble-mean averages); the confidence level is determined with a two-tailed t test. $F_{10,reg}$ is calculated from the eddy heat flux over the selected region (Appendix C)

	**A) SSW probabilistic skill**						**B) SPV deterministic skill**			**C) Regional stratospheric connections**				
region:								40-80$^\circ$ N		40-80$^\circ$ N	W Pacific	E Pacific	Pacific Sec	Eurasia
coeff:	BSS_Bn_		BSS_n_		BSS_An_		$\hbox {ACC}_{U}$	$\hbox {ACC}_F$	$\hbox {ACC}_{F(N)}$	**r**	**r**_WP_	**r**_EP_	**r**_PS_	**r**_EA_
CMCC	n.c.	(n.c.)	-0.13	(0.04)	-0.30	( -0.09)	0.48	$$\mathbf {0.49}$$	0.30	0.91$$^{**}$$	-0.05	0.75$$^{**}$$	0.40$$^*$$	0.85$$^{**}$$
MF	0.03$$^{**}$$	(0.04$$^{**}$$)	0.00	(0.15)	-0.28	( -0.07)	0.08	0.09	0.11	0.82$$^{**}$$	-0.33	0.61$$^{**}$$	0.55$$^{**}$$	0.51$$^{**}$$
ECMWF	0.06$$^{**}$$	(0.07$$^{**}$$)	-0.02	(0.17)	-0.08	(0.10$$^{**}$$)	0.13	0.08	$$\mathbf {0.28}$$	0.84$$^{**}$$	-0.22	0.53$$^{**}$$	0.26	0.71$$^{**}$$
DWD	0.03$$^{**}$$	(0.03$$^{**}$$)	-0.03	(0.12)	-0.11	(0.08$$^{**}$$)	0.37	0.22	$$\mathbf {0.43}$$	0.74$$^{**}$$	-0.35$$^*$$	0.45$$^{**}$$	0.07	0.69$$^{**}$$
UKMO	0.14$$^{**}$$	(0.14$$^{**}$$)	0.04$$^*$$	(0.19)	0.02$$^{**}$$	(0.19$$^{**}$$)	$$\mathbf {0.36}$$	$$\mathbf {0.36}$$	0.31	0.89$$^{**}$$	-0.40$$^*$$	0.68$$^{**}$$	0.19	0.64$$^{**}$$
ERA-I										0.91$$^{**}$$	0.14	0.01	0.11	0.72$$^{**}$$

$$^{**}$$/$$^*$$ significant at 95% / 90%

### Stratospheric dynamics in seasonal prediction systems

The interaction between the lower stratosphere and the polar vortex aloft is analysed in detail. Our purpose here is to improve the understanding of the role played by upward propagating waves in the seasonal variability of the stratospheric mean flow. The influence of LSWA, recall 100-hPa meridional eddy heat flux, on the SPV wind is studied: we reconstruct the wind anomaly ($\varDelta {\hat{U}}$) based on an integral of the eddy heat flux (*F* in Eq. (2)), which is obtained by adapting the theoretical results of Hinssen and Ambaum (2010) (henceforth HA, see Sect. 2.2). A linear regression between the effective SPV wind anomaly ($\varDelta {\overline{U}}$) and the integral on the eddy heat flux (*F* from $\varDelta {\hat{U}}$) allows to estimate: correlation **r**, i.e. the fraction of SPV variability that is explained by LSWA; the coefficient ${\mathcal {A}}$ in Eq. (2), i.e. the magnitude of the coupling between LSWA and the SPV (${\mathcal {A}}$ is obtained from $\varDelta {\hat{U}}\sim -{\mathcal {A}}F$, see results in the following paragraph). This approach follows Austin et al. (2003), who discussed stratospheric coupling in a previous generation of climate models, but used the theoretical approach by Newman et al. (2001) relating polar stratospheric temperature to LSWA—instead of SPV vorticity/wind anomaly to LSWA (HA/this work).

The calculation of $\varDelta {\hat{U}}$ depends on $\tau$, the radiative relaxation time in the stratosphere. In Fig. 6a, we illustrate the optimal value of $\tau$ at a pressure level of 10 hPa for reanalysis, consistent with Eq. (18) in HA ($\tau = -50 \cdot ln(\theta ) + 375$, where $\theta$ is potential temperature), by computing the correlation between the different terms in $\varDelta {\hat{U}}$ (Eq. (2)) and the vortex wind. Firstly, the analysis shows that the correlation of the vortex wind with the term $-F$ is very close to the correlation with the complete wind reconstruction $\varDelta {\hat{U}}$ (dotted and smooth line in Fig. 6a, respectively). Since the November-1st initial condition has a weak influence and correlates negatively with the vortex wind for $\tau >20$ days (triple dots), we discard its contribution and consider $\varDelta {\hat{U}}\sim -{\mathcal {A}}F$. By doing this we are assuming that the wind anomaly over DJF depends on the dynamical forcing by LSWA and not on the persistence of start-November SPV anomalies; however, we point out that a possible dependence of LSWA on the start-November SPV is not excluded, particularly in the first weeks after initialisation. The correlation of the vortex wind with $-F$ remains above 0.85 with $\tau$ varying between 40 and 100 days. Also, *F* (dotted line) represents a substantial improvement compared with simple non-weighted averages of eddy-heat-flux anomalies (dashed line). The difference between the two methods, i.e. weighted and non-weighted time averages, drops if a shorter integration window is used for *F* (dot-dashed line). This result suggests that in the subseasonal range the variability of the SPV is well approximated by a simple average over previous LSWA, but that, in the seasonal range, a long-term time integral allows the inclusion of important eddy-heat-flux contributions from over 40 days before.

A similar analysis applied to the forecast systems is displayed in Fig. 6b (top panel), showing the correlation of the vortex wind with $-F$. Correlation is at its highest for $\tau \approx$ 40 days (34 days in MF), a reduced time compared to the 56 days of the ERA-Interim peak, indicating that the model mid-latitude stratospheric flow relaxes faster towards its climatology. For further analysis, a suitable value of $\tau _{10}=45$ days is chosen for $F_{10}\equiv F(\tau _{10})$. This value of $\tau$ is depicted by the vertical dotted blue line in Fig. 6b, and corresponds to HA’s Eq. (18) set to the potential temperature 700 K. In Fig. 6b (bottom panel), the correlation dependence on the wind latitude reveals no substantial model bias. For consistency with previous analyses, we consider the variable $\varDelta {\overline{U}}_{10}^{[55-70]}$ to represent the SPV-strength/wind anomaly. In the following we study its relation to $F_{10}$, in order to describe, for each prediction system, how the SPV variability is connected to the underlying wave activity.

In Figs. 7 and 8, we provide a visualisation of the relationship between the vortex wind and the eddy-heat-flux integral through scatter plots of the daily variables and of seasonal (ensemble-mean) averages, respectively. Note that the negative value of the integral $F_{10}$ is considered. In the daily analysis (Fig. 7) the larger model sample size gives rise to smooth, statistically robust distributions against the noisy ERA-Interim plot. Nonetheless, we can compare the model distributions to the main features in ERA-Interim, such as the enhanced density of points in the upper-right sector and the skewed negative tail (topmost left panel in Fig. 7). These are generally well reproduced by the forecast systems, but intramodel variations are present: MF shows the widest distribution, contrarily to CMCC which is the most compact; ECMWF and DWD distributions reproduce closely the lower-left sector in the reanalysis scatter plot, i.e. the shift of the negative tail below the linear-regression line. Values of correlation (**r**) appear to be consistent between forecast systems and reanalysis (0.86), with MF yielding the lowest and most distant value (0.75). On the other hand, the coefficient ${\mathcal {A}}$, quantifying the coupling between LSWA and the SPV, tends to be underestimated by the forecast systems ($\sim$0.15 $\hbox {K}^{-1}$) against ERA-Interim (0.171 $\hbox {K}^{-1}$), with the exception of ECMWF (0.166 $\hbox {K}^{-1}$). We note that the amplitude of ${\mathcal {A}}$ is not linked to the climatological strength of the SPV, since all forecast systems show a reduced slope independently of the sign of the model bias (Fig. 1a).

In Fig. 7, we also include scatter plots for SSW events in DJF (pink-coloured histograms), and calculate the correlation and slope between vortex wind and eddy-heat-flux integral in the [-6,0]-day window preceding the date of SSWs. In such periods the correlation in reanalysis reduces by $\sim$25%, as does the magnitude of the coupling (also by $\sim$25%). These changes in **r** and ${\mathcal {A}}$ illustrate that during SSWs the interaction between upward propagating waves and the stratospheric flow is not trivial, in that, after a rapid weakening of westerly winds followed by an inversion, upward wave propagation is suppressed and does not determine substantial changes in the mean flow (Plumb and Semeniuk 2003; Limpasuvan et al. 2004). In the forecast systems the reductions in **r** and ${\mathcal {A}}$ in the days before SSWs are between 15 and 35%, with the smallest percentage drop shown by CMCC, exhibiting also strongest climatological SPV wind. A similar, although weaker, drop in in the coupling coefficients happens in the [0,+6]-day window following SSWs (not shown).

The seasonal analysis (Fig. 8) confirms that most of the interannual variability in the winter SPV strength can be explained by anomalies of eddy heat flux at 100 hPa, with even slightly higher values of **r** and ${\mathcal {A}}$ than in the daily analysis (Fig. 7), likely because of some filtering of noise. Note that the seasonal analysis is performed with ensemble-mean anomalies, implying that a common signal among members has been retained, but similar outcomes are obtained when considering individual model ensemble members (bottom-center panel in Fig. S5). Independently of the accuracy of the model seasonal coupling—only the correlation in UKMO and CMCC is consistent with ERA-Interim’s **r**_DJF_=0.91 (90% confidence, bootstrap as in Appendix B), the predictability of the DJF extratropical stratosphere appears to be largely dependent on the predictability of November-to-February LSWA, here represented by $F_{10}$, since the latter explains 55–83% of the SPV interannual variability.

To conclude the assessment on the wave–mean-flow interaction within the stratosphere, we quantify the skill of the wave-flux reconstruction $-{\mathcal {A}} F_{10}$ in predicting the observed DJF anomalies of vortex wind (Table 2B). In UKMO and CMCC the eddy-heat-flux integral attains skill of the same order as the direct predictions of SPV wind, while in DWD it is lower (0.22 for $F_{10}$, 0.37 for $\varDelta {\overline{U}}_{10}^{[55-70]}$). This is consistent with results in Fig. 8, where DWD showed the weakest interannual link between $F_{10}$ and the vortex wind (r = 0.74). We further explore the role of LSWA by introducing a new integral $F_{10}(\text {N})$ which considers only November eddy-heat-flux anomalies. We find that in two models (DWD and ECMWF) the November based reconstruction ($-{\mathcal {A}} F_{10}(N)$) shows higher ACC than the November–February reconstruction and than the direct wind forecast. This suggests that (1) these forecast systems perform well in November, especially in the lower stratosphere; (2) the LSWA predictions for the following season (DJF) do not provide useful information on the winter SPV. On the contrary, in other models (UKMO and CMCC) the DJF eddy heat flux appears to maximize the forecast skill (ACC_F_> ACC_F(N)_, Table 2B); MF shows an overall poor performance. Such results inspire a deeper investigation of LSWA predictions—see Sect. 3.3.

### Predictability of wave activity in the lower stratosphere

In the previous sections we analysed the predictability of the mid stratosphere (Sect. 3.1) and the link between SPV variability and LSWA (Sect. 3.2). We found prediction skill beyond persistence (damping processes) and an overall good performance of the forecast systems in capturing the link between the polar vortex and wave activity at lower levels. Likewise, it is worth highlighting the increase in seasonal stratospheric predictability and improved stratospheric dynamics, including the simulation of realistic SSW occurrence, with respect to earlier generations of seasonal forecast systems (e.g. Maycock et al. 2011; Austin et al. 2003). We now assess the prediction skill of the 100-hPa local eddy heat flux ($v^*T^*$) in the monthly (November) and seasonal (DJF) range, which appear to provide seasonal predictability for the SPV. In this way, we also add a spatial characterisation to the analysis of one-dimensional eddy heat flux (LSWA) presented up to now.

**Fig. 9:**
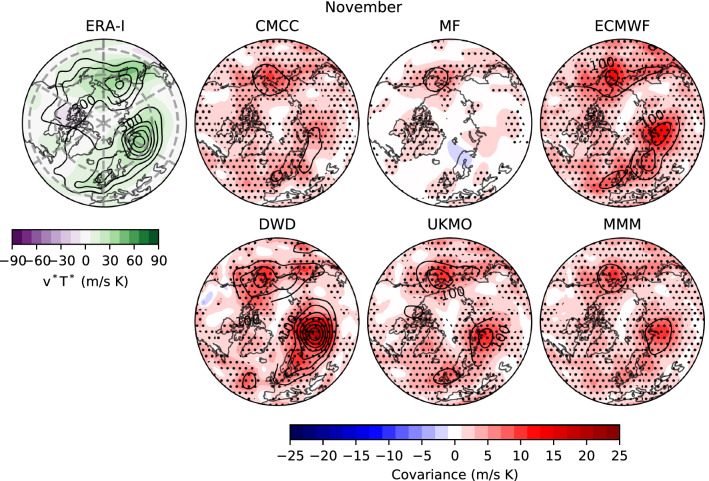
(Left) Climatology (shading) and interannual variance (contours; 150–900 (m/s K)$^2$) of 100-hPa meridional eddy heat flux ($v^*T^*$) in November for ERA-Interim. (**Right**) Covariance between ensemble-mean and (standardised) reanalysis $v^*T^*$ anomalies (shading). Black contours represent signal variance (i.e., interannual ensemble-mean variance); c.i.= 100 (m/s K)$^2$, reaching a maximum of $\sim$ 700 (m/s K)$^2$ for DWD. Statistically significant ACC for $v^*T^*$, according to a one-tailed t-test at 95% confidence level, is stippled. Results for the multi-model ensemble-mean (MMM) is also shown

The ERA-Interim climatology of $v^*T^*$ in November is displayed in Fig. 9 (shading) together with its interannual variance (contours). Also shown in Fig. 9 is the pointwise covariance between ensemble-mean and reanalysis $v^*T^*$ anomalies (shading), and the ensemble-mean interannual variance (contours; i.e. *signal variance*). The observed climatology in November exhibits strong poleward heat flux over Eurasia (Ural mountains) and western North Pacific, regions also characterised by high variability. The forecast systems show overall positive covariance and significant skill (stippling) over these areas, yielding a high signal variance, as can be inferred from the multi-model ensemble-mean (MMM). We highlight the poor performance of MF, and the skill of DWD in simulating variance values comparable to reanalysis and in predicting the observed anomalies, particularly over the Ural region; the good performance is probably linked to DWD’s ability to forecast November SSWs (Fig. S4).

**Fig. 10:**
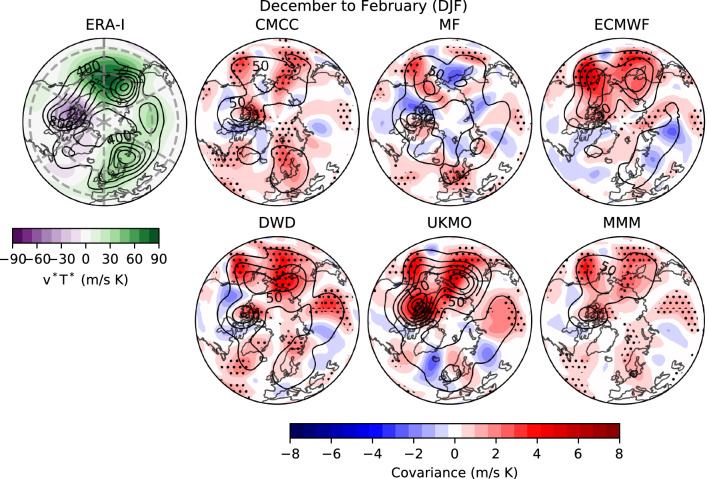
As Fig. 9, but for DJF seasonal-mean. Interannual variance for ERA-Interim ranges in 200–1200 (m/s K)$^2$. Signal variance is shown with a c.i. = 25 (m/s K)$^2$, with a maximum of $\sim$ 200 (m/s K)$^2$ for UKMO. Note the different colour scale with respect to Fig. 10

The climatology and variability of $v^*T^*$ in DJF (Fig. 10) strengthens over the North Pacific, while over Eurasia it shifts and splits in two centres of action, a weak one over central Eurasia and a strong one around the Scandinavian Peninsula. ERA-Interim also shows enhanced negative heat flux and more variability over northern Canada, as compared with November. The forecast systems lose most of their skill in DJF, but still attain significant positive covariance with reanalysis in the Pacific sector, over western and eastern North Pacific as well as over northern Canada, where they also simulate substantial signal variance (see MMM). We stress the good performance of ECMWF and UKMO in simulating and predicting $v^*T^*$ variability in these regions, although for ECMWF this does not translate into forecast skill of the SPV (Fig. 3). In the Eurasian sector, unpredictable variability (Weisheimer et al. 2019; Kim et al. 2012) causes a weak ensemble-mean signal variance, which, added to model diversity in the location of the anomalies, leads to a weak multi-model signal variance (black MMM contours in Fig. 10). Yet, it is to note that the three forecast systems that better capture the two centres of action over Eurasia, namely CMCC, DWD and UKMO (reflected in the MMM), are those showing the highest prediction skill of the SPV (Figs. 2b, 3). Again, MF shows the worse performance among the C3S forecast systems in both the Pacific and Eurasian sectors.

**Fig. 11:**
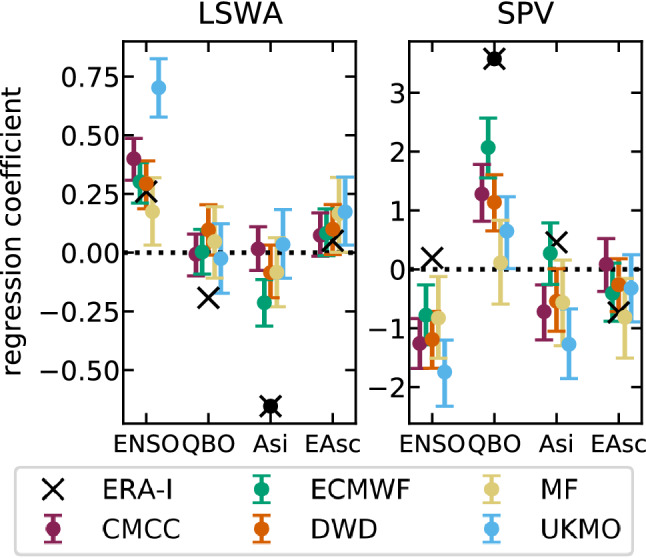
Regressions of DJF lower-stratosphere wave activity ($[v^*T^*]$, left) and polar vortex wind (${\overline{U}}_{10}^{[55-70]}$, right) onto potential sources of predictability, for ERA-Interim and the ensemble-mean forecasts. The potential sources, taken from reanalysis, are ENSO, the QBO, Arctic sea-ice extent (Asi), Eurasian snow cover (EAsc); ENSO and the QBO are considered in winter (DJF), while Asi and EAsc in autumn (ON). Error bars represent the 5th and 95th percentiles of model regression slope—the distribution is calculated using a bootstrap (Appendix B, for slope instead of correlation). Statistical significance at 90% confidence level according to a two-tailed t-test is indicated for ERA-Interim with full black circles

Next we investigate the contribution of potential sources of predictability to the seasonal prediction skill of the stratosphere (Figs. 2, 10), specifically DJF ENSO (Garfinkel and Hartmann 2008), DJF QBO (Baldwin et al. 2001), ON Arctic sea-ice extent (Jaiser et al. 2013) and ON Eurasian snow cover (Cohen et al. 2014). A linear regression is computed between LSWA/SPV and the observed DJF/ON signals—standardised for comparisons; reanalysis and model results are displayed in Fig. 11. Here, we do not take into account how C3S models represent the aforementioned signals and the arising processes, as it is beyond the scope of the paper.

In ERA-Interim, the strongest and most significant link with LSWA is found for Arctic sea-ice extent; however, it is not robustly associated with changes in the SPV, although the sign is consistent—less wave activity is related to a reinforced polar vortex. The forecast systems show overall a weak, sometimes inconsistent, relationship between Arctic sea-ice extent and the stratospheric circulation. Both ERA-Interim and the forecast systems yield a weak and largely non-significant link between Eurasian snow cover and LSWA/SPV, in agreement with the decay of the connection between this predictor and the polar vortex during recent decades (Henderson et al. 2018). The QBO, on the other hand, shows the strongest and most significant link with the observed SPV. It is simulated correctly, but with a weak amplitude, by the forecast systems, with the exception of MF. The relationship with the SPV does not relate to statistically significant changes of LSWA in either ERA-Interim or the forecast systems. In the context of the Holton-Tan effect where the QBO can modulate the deflection of upward-propagating waves, this could be explained by the absence of a clear link of the QBO with anomalous wave injection (e.g. Holton and Tan 1980), or better by the complex latitudinal dependence of the anomalous wave injection into the stratosphere (Garfinkel et al. 2012b; White et al. 2015, 2016). Finally, the most robust signal simulated by the forecast systems is that associated with ENSO, with significant anomalies in LSWA and SPV; in the case of El Niño, for instance, more wave activity is related to a weaker polar vortex. However, in ERA-Interim the relationship between ENSO and the SPV/LSWA (see Domeisen et al. 2019, for a review) is not significant between 1993–1994 and 2016–2017. This result is in agreement with the decay of the teleconnection in recent decades (Garfinkel et al. 2019).

**Fig. 12:**
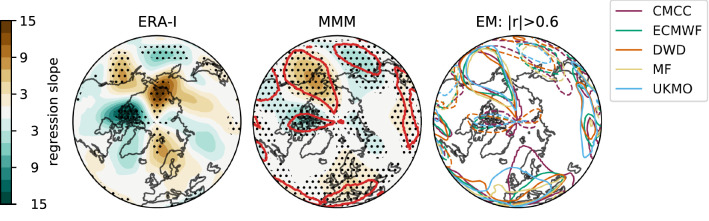
Regression maps of 100-hPa meridional eddy heat flux ($v^*T^*$) anomalies in DJF on the observed (standardised) Niño3.4 index, for ERA-Interim (left) and the multi-model ensemble mean (MMM, center). Statistically significant areas according to a two-tailed t test at 90% confidence level are stippled, while red contours for MMM enclose regions where all five systems agree on the sign of the regression slope. The map on the right—EM—shows individual ensemble-mean regressions; regions with correlation greater (smaller) than 0.6 (− 0.6) are indicated with full (dashed) contours

**Fig. 13:**
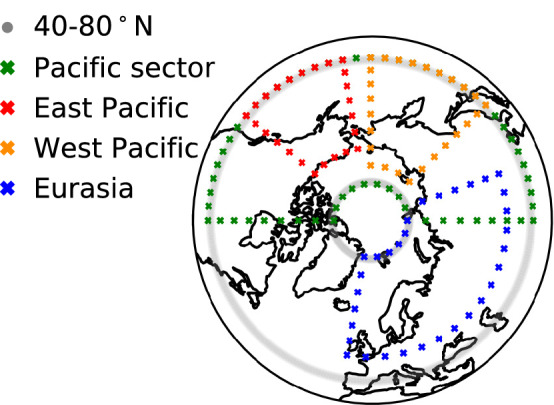
Regions corresponding to 40–80$^\circ$ N (grey), Pacific Sector (green), West Pacific (orange), East Pacific (red), and Eurasia (blue), as in analyses of Table 2C, Fig. S5

To further explore the influence of ENSO on lower-stratospheric wave activity, regression maps of $v^*T^*$ onto the observed Niño3.4 index are shown in Fig. 12 for ERA-Interim (left), the MMM (center) and each individual forecast system (right). A salient and robust signature is a dipole-like pattern in the Pacific sector, with positive anomalies over the eastern North Pacific, which extend to polar latitudes, and negative anomalies over the western North Pacific. This ENSO-related dipole pattern of eddy heat flux is in agreement with results from a recent multi-model sensitivity experiment prescribing El Niño SST forcing (Palmeiro et al., submitted). Another robust signal across forecast systems and ERA-Interim is the negative eddy-heat-flux anomaly over northern Canada. These three centers of action in $v^*T^*$ are reasonably well predicted by four forecast systems (CMCC, ECMWF, DWD, UKMO; Fig. 10). More diversity among forecast systems is found in ENSO-related $v^*T^*$ anomalies over Europe; this signature is lost in reanalysis (Fig. 12).

In a final attempt to shed light on the connection between 100-hPa eddy heat flux and the SPV, we assess how regional $v^*T^*$ provides forcing to the variance of the SPV wind. Apart from the Pacific sector, linked to ENSO as discussed above and in Orsolini et al. (2009), the Eurasian sector is the other key area modulating wave injection into the stratosphere. Its wave activity has been shown to trigger AO- and NAO-like variability (e.g. Kuroda and Kodera 1999; Takaya and Nakamura 2008), and to precede SSWs (e.g. Limpasuvan et al. 2004; Orsolini et al. 2011; Karpechko et al. 2018). We focus on four regions described in Appendix C: *East Pacific*—EP and *West Pacific*—WP, included in the larger *Pacific Sector*—PS, and *Eurasia*—EA. Similarly to Sect. 3.2, we construct time integrals on *regional* eddy-heat-flux anomalies and we assume that the vortex wind anomaly is forced independently by each regional wave forcing—the new variable $F_{10,reg}$ is compared with the SPV wind anomaly (Table 2C). Note that DJF (ensemble-mean) averages are considered, comparable to Fig. 8. Firstly, it is worth stressing that all $F_{10,reg}$ exhibit a weaker correlation with the SPV variability than $F_{10}$, based on the 40–80$^\circ$ N eddy heat flux; this applies to both reanalysis and the forecast systems. Secondly, in ERA-Interim the total LSWA is largely dominated by the $v^*T^*$ contribution from Eurasia (**r**_EA_=0.72), with minor and statistically non-significant contributions from the Pacific sector (PS and its sub-regions). Such dominant role of the Eurasian eddy heat flux is captured by all the forecast systems except MF (**r**_EA_ = 0.51–0.85 vs **r**_PS_ = 0.07–0.55). However, the forecast systems tend to overestimate the contribution from the eastern North Pacific, with **r**_EP_ = 0.45–0.75 against **r**_EP_ = 0.01 in ERA-Interim. They also exhibit an anticorrelation between SPV anomalies and $v^*T^*$ over the western North Pacific ($F_{10,WP}$), which is however not significant. The results for the Pacific sector are altogether consistent with those discussed above for ENSO; in particular, the dipole of $v^*T^*$ in the North Pacific (Fig. 12) and the overestimation of the ENSO influence on LSWA and SPV wind by forecast systems (Fig. 11). The regional analysis applied to individual ensemble members over different time ranges (Fig. S5) confirms the model overestimation of the East-Pacific link, while the Eurasian link is weak compared with reanalysis. It also appears that the relation of the SPV with the Pacific eddy heat flux is stronger at time scales up to one month, while for Eurasia it is strongest in the seasonal scale; models do not capture the strengthening of the Eurasia-SPV relation at the seasonal range.

## Discussion

Winter variability and prediction skill of the Northern-Hemisphere stratosphere are investigated in five state-of-the-art seasonal forecast systems initialised in November. In the mean flow at 10 hPa we identify three latitudinal regions: the *tropics*, found to be highly predictable due to the initialisation of the QBO phase; the *subtropics*, characterised by a low interannual variability that is not predicted by C3S hindcasts; the *extratropics*, where the stratospheric polar vortex is variably predicted among the forecast systems.

The seasonal hindcasts show a realistic variability in the extratropical stratosphere, in terms of magnitude of anomalies, latitude of maximum variability and occurrence of SSWs. This is a considerable improvement compared with previous model generations (c.f. Maycock et al. 2011). Moreover, a subgroup of systems (CMCC, DWD and UKMO) is able to capture the predictable component of the extratropical signal (cf. Figs. 2a, b), and is substantially more skilful than empirical forecasts computed with October/November ERA-Interim anomalies—subgroup ACC is $\sim 0.5$, see Figs. 2b, 3 and 4. Similar results are found at lower levels, i.e. 100–30 hPa (Fig. S2). Butler et al. (2016) displayed a similar SPV skill for a different, yet intersecting, set of seasonal prediction systems, and emphasised the higher stratospheric skill of high-top models compared with low-top ones (their Figure 1(c)). We add that among high-top models no clear relationship appears to exist between stratospheric resolution—vertical and horizontal—and skill (cf. Table 1; Fig. 2). Nonetheless, a finer grid favours a realistic frequency of the stratospheric events that have the greatest impact on the troposphere, i.e. SSW events (Fig. 5; Charlton-Perez et al. 2013).

As expected, the strength of the DJF vortex wind in ERA-Interim changes depending on the SSW occurrence throughout the season (multiple/no events, see Fig. 4, bottom panels). To a lesser extent, also the SPV simulated by forecast systems is sensitive to the observed SSW occurrence, suggesting that some of the winter skill may derive from predictions of the number of SSW events in DJF. We assess the probabilistic predictability of DJF SSWs by devising category (tercile-oriented) forecasts. The associated score (BSS) reveals that the forecast systems predict skilfully the absence of SSWs over winter (Table 2A); category forecasts of multiple-SSW winters are less effective. This finding deserves further investigation, as it prompts the existence of windows of opportunities to predict the absence of winter SSWs—strong vortex conditions—from late autumn, e.g. record-breaking 2019/20 vortex (Lee et al. 2020b; Lawrence et al. 2020). Similar conclusions hold for the predictability of vortex events at shorter time scales (Domeisen et al. 2020a). The high skill in predicting the strong-vortex state is expected to derive from the increased predictability of the lower stratosphere in periods characterised by weak wave activity, rather than in those characterised by the intense, noisy wave activity inducing a weak vortex. Such periods correspond to the linear and non-linear regimes in the relation between the vortex wind and the eddy-heat-flux integral, respectively (Fig. 7, details in the following paragraph).

The variability of the SPV is indeed closely connected to LSWA, which propagates upwards and breaks in the strong mean flow, slowing down the vortex. Such a link is typically captured by forecast systems on daily time scales (less so by MF), as we assess by applying the theoretical arguments in HA (Fig. 7). The exercise demonstrates that, throughout DJF, the weighed time integral of LSWA co-varies with the strength of the SPV. The linear relationship fails in the days immediately before (Fig. 7) and after (not shown) an SSW event, when the decelerated flow responds weakly to the suppression of the upward wave flux (Limpasuvan et al. 2004; Plumb and Semeniuk 2003). We report two biases common to the C3S multi-model, namely the weak impact of eddy heat flux on the vortex, and the short radiative relaxation time scales in the mid stratosphere (i.e. the time required for wave-induced SPV anomalies to decay, Fig. 6a); both are consistent with Maycock et al. (2011). The two features may be linked, since reduced impact of LSWA is liable to cause faster vortex relaxation, as reflected in MF which exhibits the weakest daily wave–vortex coupling and the fastest radiative relaxation among the C3S system. The interannual LSWA–SPV relation is captured by the climate models, with 50-to-83% (83% in ERA-Interim) of the interannual SPV-wind variability explained by the winter LSWA (Fig. 8).

In short, our study demonstrates that seasonal climate anomalies and their predictability are explained by a modulation of the 100-hPa eddy heat flux, interpretable as a modulation of upward propagating planetary waves. We distinguish two separate LSWA time scales contributing to the seasonal prediction skill of the SPV. In two forecast systems (DWD and ECMWF) most of the winter skill depends on November LSWA; conversely, in CMCC and UKMO the winter skill benefits from the prediction of wave activity in DJF (Table 2B). The difference in the behaviour of LSWA is particularly wide when considering DWD and CMCC, models which also exhibit opposite SPV biases (Fig. 1). In CMCC the strong linearity between the vortex and LSWA flux resembles the reanalysis. Strong linearity and high winter skill are possibly linked to the limited amount of SSWs in the model, corresponding to reduced high-frequency noise in the stratospheric layers (see e.g. Fig. 7); further analyses are needed to prove such an hypothesis. This system also produces a signal-to-noise paradox in the stratosphere, a feature that is generally strong in the tropospheric Euro-Atlantic sector (Siegert et al. 2016; Scaife and Smith 2018). Regarding DWD, we lay emphasis on the realistic signal variance and the significant skill in the lower stratosphere during November, in particular over the Eurasian continent (Fig. 9; Song et al. 2020), which appear to account for the remarkable forecasts of November SSWs and for the significant predictive skill in the winter SPV.

The contribution of potential sources of seasonal predictability is explored. ENSO allows for enhanced eddy-heat-flux seasonal prediction skill over the Pacific sector. However, the stratospheric response to ENSO in prediction systems is strong and linear compared with the observed response, which is characterised by an asymmetric impact of opposite ENSO phases (not shown, see also Butler and Polvani 2011; Garfinkel et al. 2019; Domeisen et al. 2019). This bias is consistent with findings by Garfinkel et al. (2019), who revealed a recent weakening of the ENSO–SPV teleconnection that is not captured by climate models. The weakening is thought to be induced by the increasing importance of the Eurasian eddy heat flux (Garfinkel et al. 2019; Cohen et al. 2019; Peings 2019; White et al. 2018; Lee et al. 2020a; Domeisen et al. 2020b). We also report that a weak connection of the QBO with the SPV in the C3S multi-model may provide some vortex predictability, but a comparison with the strong observed connection confirms issues in the modelling of the Holton–Tan mechanism (Garfinkel et al. 2018; Butler et al. 2016). On the basis of the results by Rao et al. (2020) and Butler et al. (2016), showing that climate models fail to simulate the mid-stratospheric response to the QBO in the 20–40$^\circ$ N region (see also our Fig. S2), we hypothesise that a better model performance in the subtropical stratosphere may support the QBO signal in the extratropics (for details on the importance of this region see Garfinkel et al. 2012b); we encourage further research on the matter. Moreover, our analysis does not detect any significant role of Eurasian snow cover and sea ice for the predictability of the stratosphere (for an extensive treatment of Eurasian snow cover in the C3S models see Ruggieri et al., in review).

A closer analysis of model predictions in the lower stratosphere reveals that the stratospheric predictability from ENSO does not generally translate into SPV skill, since, over the hindcast period, it is the wave injection from the Atlantic sector (Eurasia), not that from the North Pacific, that most correlates with the observed vortex variability (Table 2C; Fig. S5; Zhang et al. (2016)). We specify that these results concern the average LSWA–SPV relation and do not rule out periods when the vortex is modulated by the Pacific wave activity, nor an influence of the Pacific troposphere on the Eurasian lower stratosphere. Yet all C3S prediction systems, in particular ECMWF, exhibit a low skill in forecasting the seasonal 100-hPa eddy heat flux over North Atlantic and Eurasia: ECMWF’s performance in the Atlantic sector is probably limited by the low skill in the underlying troposphere (Fig. 10; Dobrynin et al., submitted; Kim et al. 2012; Baker et al. 2018), and reflects in the poor SPV predictions; by contrast, the C3S forecast systems which exhibit the highest skill in the Atlantic-Eurasian sector also perform better in the stratosphere [cf. Dobrynin et al. (submitted) and Fig. 3], corroborating the idea that the stratospheric skill feeds on the prediction of wave injection, in particular that generated by the Eurasian tropospheric flux (Orsolini et al. 2018; Peings 2019; Schlichtholz 2019). Just as the representation of the stratosphere is known to impact the ability to forecast the mid-latitude tropospheric flow (Nie et al. 2019; Stockdale et al. 2015; O’Reilly et al. 2019), here the prediction of tropospheric variability appears to regulate the seasonal forecast skill of the SPV. Analogous results seem to hold at shorter time scales, e.g. Lee et al. (2020a); Lehtonen and Karpechko (2016); Domeisen et al. (2020b).

An exception to the average Eurasian–SPV link is found in individual winters, e.g. in 2019/20 winter, when a strong North-Pacific forcing linked to the Indian Ocean Dipole induced an anomalous seasonal SPV strength (Hardiman et al. 2020). The North-Pacific anomalies were well captured by C3S seasonal models, leading to a remarkable prediction of the SPV (Lee et al. 2020b). Consistently with our own hypothesis, Lee et al. (2020b) evidenced the interconnection between stratospheric and tropospheric forecasts and noticed a lacking connection in MF, giving the least skilful predictions for January–February–March 2020. Here in MF we detect a reduced vertical coupling within the stratosphere, i.e. between LSWA and SPV (Fig. 7).

After discussing in depth the seasonal predictions of the winter SPV, we suggest two procedures aiming to improve their performance. Firstly, recall how November LSWA is important for stratospheric predictions of the following winter. Increased data assimilation in the upper troposphere and in the stratosphere is expected to improve the accuracy of initial conditions and, presumably, to have a positive impact on stratospheric predictions in the subseasonal range (Noguchi et al. 2016). The performance for the season starting one month after initialisation would also rise, since good subseasonal forecasts of the 100-hPa eddy heat flux are seen to contribute to the seasonal SPV skill (e.g. in DWD, see Table 2B). Secondly, the work by Dobrynin et al. (2018), studying a teleconnection-based subsampling approach to seasonal forecasting, inspires the application of similar techniques to the stratosphere. The same approach, or others, especially designed for the stratospheric vortex, may yield attractive results. Improved seasonal predictions of the stratosphere and its wave activity are then expected to provide additional information regarding the probability of SSW occurrence throughout winter.

## Conclusions

In this work we have assessed the variability and prediction skill of the winter stratosphere in the C3S seasonal prediction systems initialised in November.

- Three out of five systems show significant skill for the winter stratospheric polar vortex, proving that dynamical forecasts can predict the winter mid-latitude stratosphere better than persistence forecasts based on late-autumn anomalies (Fig. 3). Part of the skill could derive from the ability in predicting (probabilistically) the absence of SSWs (Table 2); incidentally, one model (DWD) shows good predictions of November SSWs (Fig. S4).
- In this set of high-top forecast systems, the seasonal skill of the polar vortex does not appear to depend on resolution. A finer horizontal and vertical grid spacing, nonetheless, improves the representation of stratospheric processes, e.g. SSW frequency (Fig. 5). Indeed we note advances in the simulation of vortex strength and variability with respect to previous generations of seasonal prediction systems (Fig. 1).
- In reanalysis, December-to-February anomalies in vortex strength are largely explained by November-to-February wave activity in the lower stratosphere (Fig. 8). This implies that (1) the representation of the stratospheric wave–mean-flow interaction and (2) the prediction of the 100-hPa eddy heat flux from November are equally important to forecast the strength of the polar vortex. The wave–mean-flow interaction is well represented by the forecast systems (Fig. 7), yet the radiative relaxation of the simulated polar vortex is faster than in reanalysis (Fig. 6); this bias might have important implications for predictability and deserves targeted research.
- The models’ performance in predicting the 100-hPa eddy heat flux is overall high in November, then decays in winter with residual skill in the Pacific sector (Figs. 9, 10); however, over this hindcast period, North Pacific eddy heat flux does not appear to drive the interannual variability of the stratospheric polar vortex. Conversely, Eurasian eddy heat flux, generated chiefly by the tropospheric flow, shows strong covariance with the polar vortex in both reanalysis and the forecast systems (Table 2).
- Over this hindcast period, the observed interannual variability of the stratospheric polar vortex is significantly affected by the QBO, and less so by ENSO. A subset of forecast systems capture the QBO teleconnection (weaker than in reanalysis), while ENSO provides predictability of the 100-hPa eddy heat flux in the Pacific sector, but its stratospheric signature is overestimated by the forecast systems (Figs. 11, 12).

On the basis of the well-known relationship between stratospheric and tropospheric circulations, we speculate that the two-way coupling does not only apply to variability but also to predictability, and this could be particularly relevant for the North Atlantic-Eurasian region; with regard to the stratospheric influence on the tropospheric flow, we encourage further investigation on the downward-coupling mechanisms in the C3S multi-model.

How will operational seasonal forecasts predict the winter stratosphere in the near future? Enhanced data assimilation above the mid troposphere is thought to improve predictions of the wave modulation in the lower stratosphere, leading to additional seasonal forecast skill for the stratospheric polar vortex. On the other hand, process-oriented subsampling approaches may allow for further skill from available ensemble predictions. If these methods prove successful, we expect an important advance in predicting the probability of winter SSWs.

### Supplementary Information

Below is the link to the electronic supplementary material.

Supplementary material 1 (pdf 994 KB)

## Data Availability

Data for ERA-Interim and Copernicus Climate Change Service seasonal multi-system forecasts was obtained from the Copernicus Climate Data Store (https://cds.climate.copernicus.eu).

## Appendix 1: Equation for upward coupling

Starting from the *Eulerian zonal mean quasi-geostrophic theory* (Holton and Hakim 2013, Sect. 10.2.1), Hinssen and Ambaum find the following equation for polar cap potential vorticity at fixed pressure-height, north of a given latitude $\phi _0$

9 $$\begin{aligned} \left\langle {\overline{q}} \right\rangle _{\phi _0}(t) = \left\langle {\overline{q}} \right\rangle _{\phi _0}(0) \; e^{-t/\tau } + \int _0^t \frac{\left\langle {\overline{q}} \right\rangle _{\phi _0,R} (t^\prime )}{\tau } \; e^{-\frac{t-t^\prime }{\tau }} \mathrm {d}t^\prime - {\mathcal {A}} \int _0^t [v^*T^*]_{\phi _0} (t^\prime ) \; e^{-\frac{t-t^\prime }{\tau }} \mathrm {d}t^\prime , \end{aligned}$$

where overbar indicates the zonal mean, square brackets indicate area-weighed mean, *q* stands for potential quasi-geostrophic vorticity, $v^* T^*$ for meridional eddy heat flux at 100 hPa, $\tau$ for radiative relaxation time in stratosphere (function of pressure level), $\left\langle {\overline{q}} \right\rangle _{\phi _0,R}$ is the radiative equilibrium value.

From the equation for *zonal-mean quasi-geostrophic potential vorticity* (Holton and Hakim 2013, Eq. (10.23)) we can write the deviation from climatology as

10 $$\begin{aligned} \varDelta {\overline{q}} = - \frac{\partial \varDelta {\overline{U}}}{\partial y} + f_0 \rho _0^{-1} \frac{\partial \varDelta \overline{\rho _0 \theta _e / \theta _{0z}}}{\partial z} \end{aligned}$$

with $\theta _{0z}$ reference potential temperature profile, $\theta _e$ departure from the reference, *y* northward distance and *U* zonal component of wind. The second term on the RHS of Eq. (10) can be neglected in stratosphere, due to the prevalent adiabaticity (Andrews et al. 1987; Newman et al. 2001), so that

11 $$\begin{aligned} \varDelta {\overline{q}} \approx - \frac{\partial \varDelta {\overline{U}}}{\partial y}. \end{aligned}$$

According to the approximation, the anomaly of stratospheric polar cap vorticity at a given pressure level, north of latitude $\phi _0$ is

12 $$\begin{aligned} \varDelta \left\langle {\overline{q}}\right\rangle _{\phi _0} = \int \limits _{\phi _0}^{90^\circ \text {N}} \varDelta {\overline{q}} \; \mathrm {d}y \approx - \int \limits _{\phi _0}^{\text {90}^\circ \text {N}} \frac{\partial \varDelta {\overline{U}}}{\partial y} \; \mathrm {d}y = + \varDelta {\overline{U}}_{\phi _0} \end{aligned}$$

because zonal-mean zonal wind is always null at the Poles. By combining Eqs. (9) and (12) we obtain the final approximation

13 $$\begin{aligned} \varDelta {\overline{U}}_{\phi _0} (t) \approx&\, \varDelta {\overline{U}}_{\phi _0} (0) \; e^{-t/\tau } - {\mathcal {A}} \, F_{\phi _0} (t)\nonumber \\\equiv& \varDelta {\overline{U}}_{\phi _0} (0) \; e^{-t/\tau } \nonumber \\&\quad - {\mathcal {A}} \int _0^t \varDelta [v^*T^*](t^\prime ) \; e^{-(t-t^\prime )/\tau } \mathrm {d}t^\prime , \end{aligned}$$

where we have neglected the radiative contribution, small compared with the heat-flux integral, defined as $F_{\phi _0}$, and we take the average of $v^*T^*$ in the region where wave activity propagates towards the stratospheric vortex (40-80$^\circ$ N, as in Hinssen and Ambaum (2010)).

## Appendix 2: Bootstrap for correlation

*Bootstrap resampling* consists in reproducing an unknown distribution from a set of independent values (Davison and Hinkley 1997)—in this case 1000 correlation values per system, obtained from the comparison between 1000 samples of the model time series and the observed sequence. The model and reanalysis time series are identified as $\{{\mathbf {m}}_i\} = \{m_{y,i}\}$ and ${\mathbf {o}}=\{o_y\}$, with year $y \in [1,24]$ and sample $i \in [1,1000]$. Each yearly ensemble-mean $m_{y,i}$ is calculated using the Monte–Carlo algorithm

$$\begin{aligned} m_{y,i} = \frac{1}{n_m}\sum _{j\in \{r_i\}} m_{y,j} \end{aligned}$$

where $n_m$ is the size of the ensemble, *j* indicates an individual ensemble member, $\{r_i\}$ is the *i*th sequence of $[n_m]$ indices extracted randomly with replacement from the set $\{1\ldots n_m\}$. Thus, the *i*th correlation value is equal to

$$\begin{aligned} \text {r}_i = \frac{\rho ({\mathbf {m}}_{i},{\mathbf {o}})}{\sigma ({\mathbf {m}}_{i}) \cdot \sigma ({\mathbf {o}})}. \end{aligned}$$

To apply *bootstrapping* we assume model simulations within the same year to be independent, requirement that is satisfied due to small randomised perturbations of the initial conditions.

## Appendix 3: Regional eddy heat flux

Figure 13 shows the areas selected for the analysis of regional eddy heat flux in Table 2C: *East Pacific* [EP: 190–230$^\circ$ E; 40–70$^\circ$ N], *West Pacific* [WP: 135–180$^\circ$ E; 40–75$^\circ$ N], *Pacific Sector* [PS: 90–270$^\circ$ E; 40–80$^\circ$ N] and *Eurasia* [EA: $10^\circ$ W–110$^\circ$ E; 50–80$^\circ$ N]. The average regional quantity is obtained as the mean on the grid points, each weighed by the cosine of the correspondent latitude.
