# Supplementary material for: Seasonal prediction of the boreal winter stratosphere
Source: Clim Dyn. 2021 May 18;58(7-8):2109–30. doi: 10.1007/s00382-021-05787-9 (PMC9012732; doi:10.1007/s00382-021-05787-9)
Supplement: Supplementary file 1 — Supplementary material 1 (pdf 994 KB) [file 382_2021_5787_MOESM1_ESM.pdf]

Climate Dynamics

Supplementary material

Seasonal prediction of the boreal winter stratosphere

Alice Portal · Paolo Ruggieri · Froila M. Palmeiro · Javier García-Serrano · Daniela I.V. Domeisen · Silvio Gualdi

A. Portal

Department of Earth and Environmental Sciences, Università degli Studi di Milano-Bicocca, Milano, Italy

E-mail: a.portal@campus.unimib.it

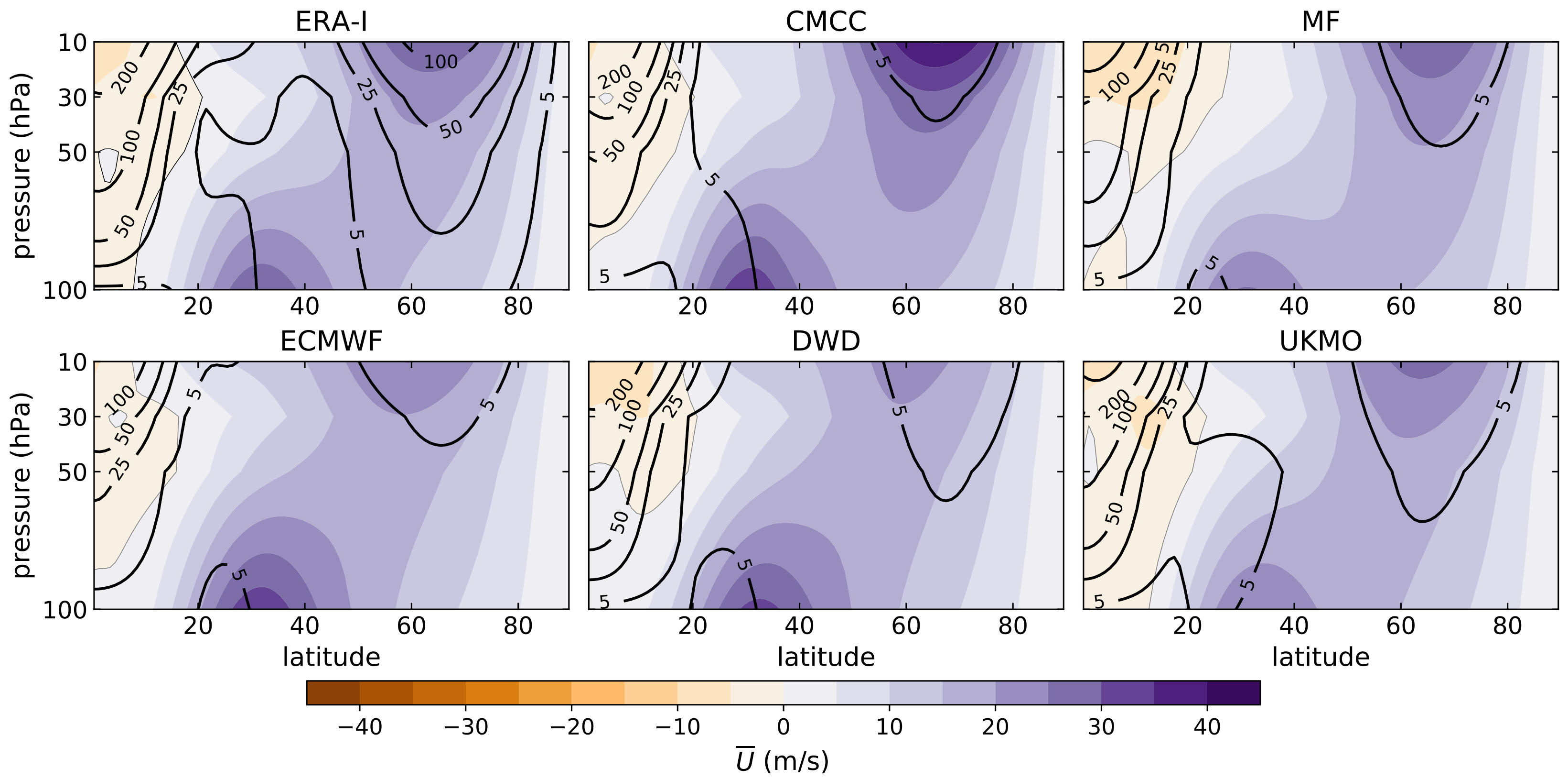

Fig. S1 Climatology of DJF zonal-mean zonal wind at pressure levels from 100 to 10 hPa. Thick black contours show interannual (ensemble-mean) variance.

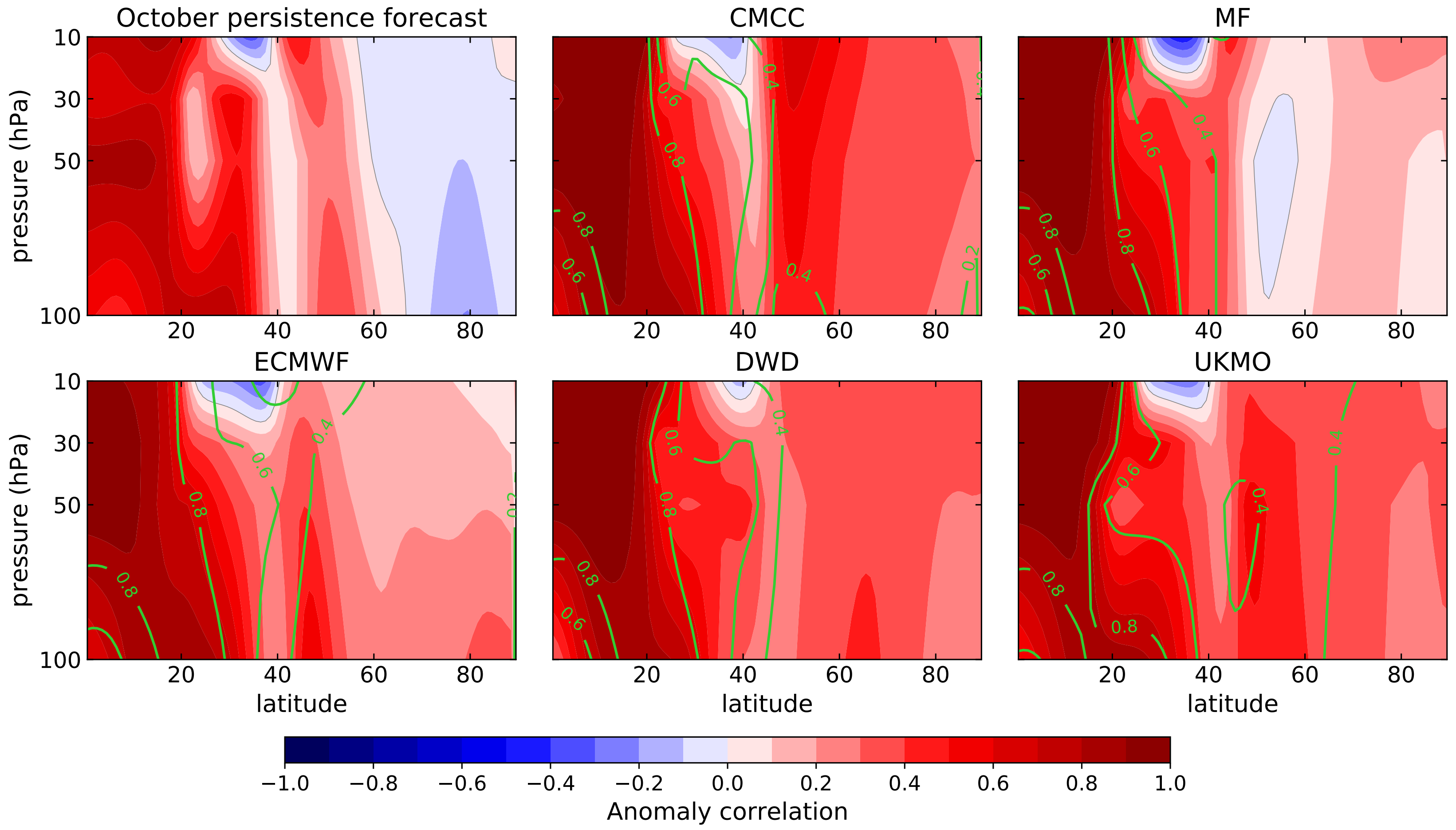

Fig. S2 Anomaly correlation coefficient between ensemble mean and reanalysis DJF zonal-mean zonal wind, at pressure levels from 100 to 10 hPa. Green contours show square root potential predictability (σ_s/σ_t). The persistence forecast based on October ERA-Interim anomaly is shown in the top-left panel as a benchmark for dynamical forecasts.

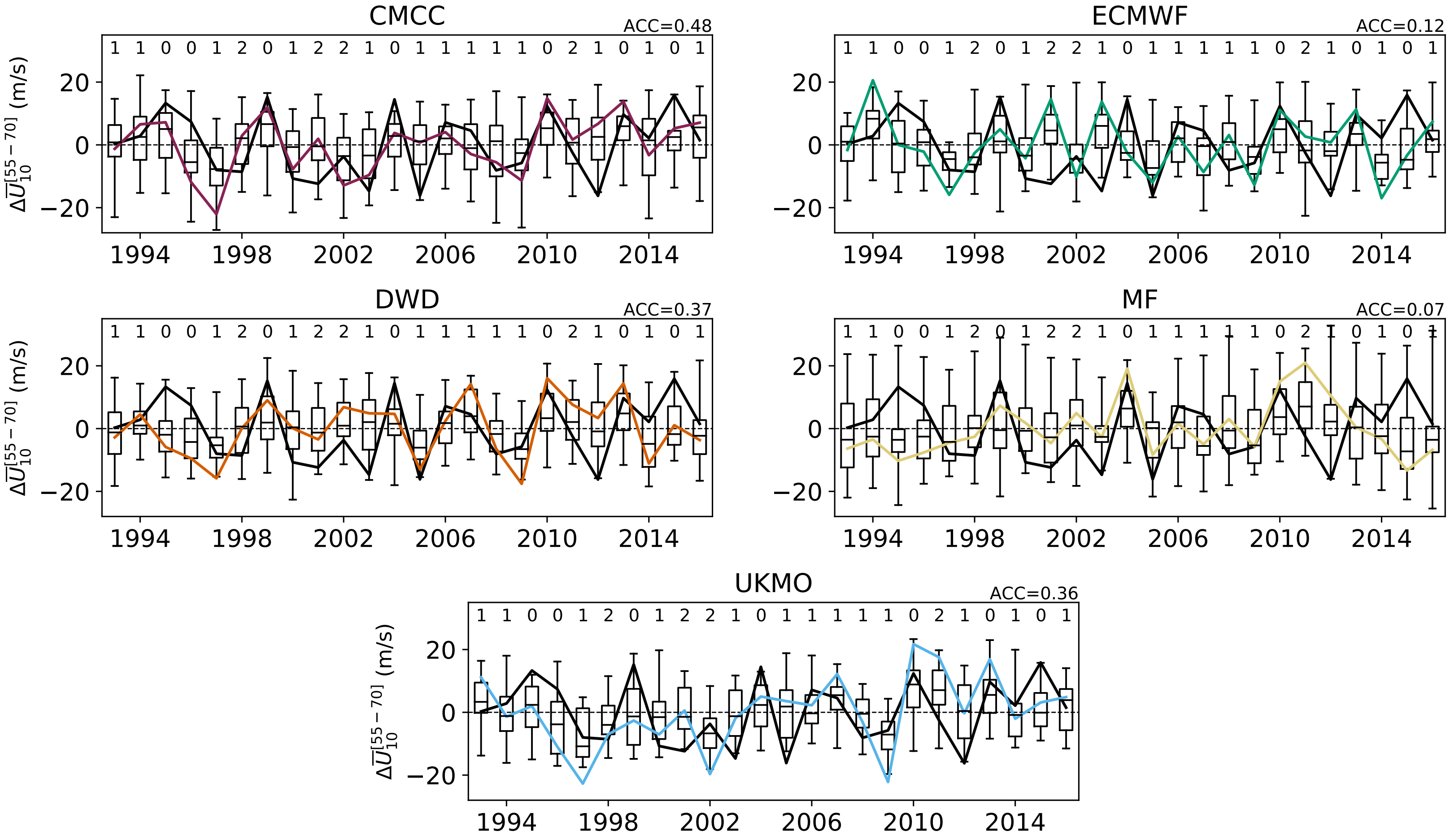

Fig. S3 Time series of DJF $\overline{U}_{10}^{[55-70]}$ anomaly for ERA-Interim (black line) and for model ensemble mean (coloured line), aligned with the number of SSWs observed for each year between December and February (numbers on the top strip). The amplitude of the ensemble-mean anomaly is multiplied $\times 3$ to appreciate interannual variations, while anomaly correlation between ensemble-mean forecast and reanalysis is indicated top-right of each plot. Behind the time series, boxplots illustrate the distribution of the model ensemble hindcast for each year: the horizontal line is the median, the box is the interquartile range, the whiskers cover the entire range of the distribution.

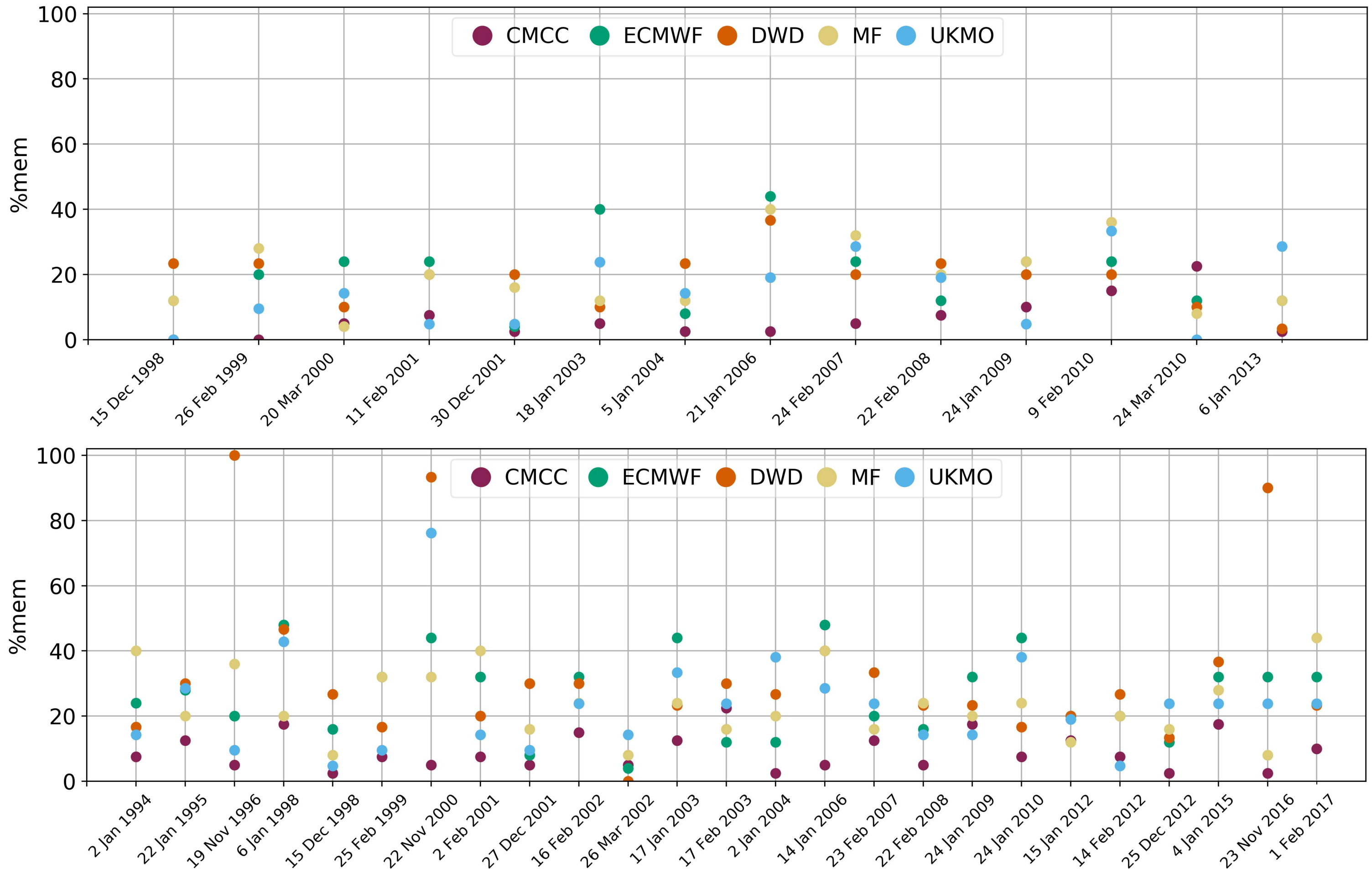

Fig. S4 For each SSW date detected in ERA-I (x-axes), coloured dots in the vertical indicate the percentage of members of each model that predict it in a window of ±10 days using (top) 60N and (bottom) 55_70N to define SSWs (see Sect. 2.2 for details).

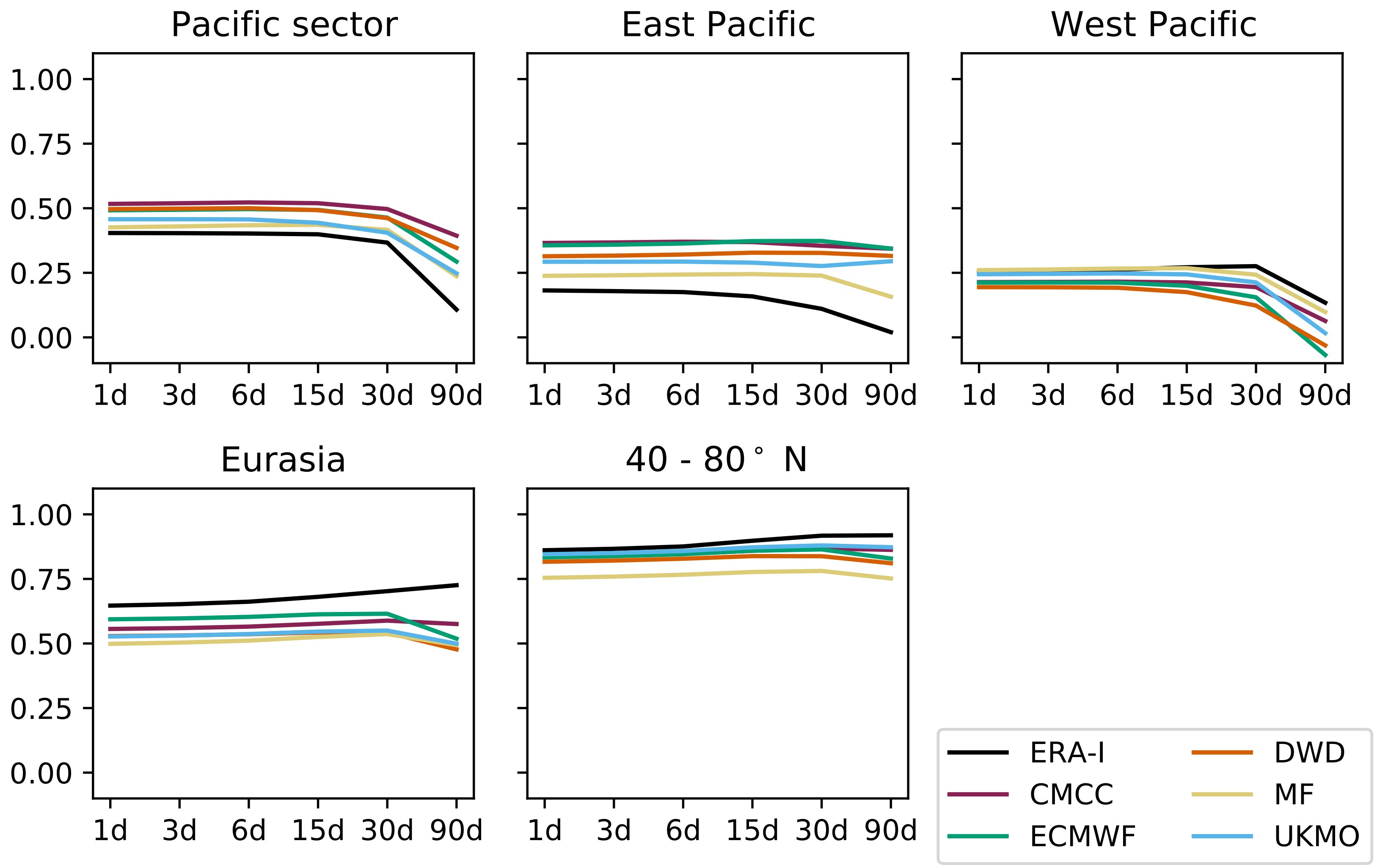

Fig. S5 Correlation between $\Delta\overline{U}_{10}^{[55-70]}$ and $-F_{10,reg}$ (full $-F_{10}$ in the central panel, bottom row) over DJF applying time filters, i.e. averages, from the daily (1d) to the seasonal (90d) range. Results are shown for ERA-Interim and for forecast systems—individual ensemble members are considered.
